# Myocardial fibrosis and glycolysis: a bibliometric study and visualization analysis (2000–2024)

**DOI:** 10.1186/s12967-026-08005-4

**Published:** 2026-03-17

**Authors:** Qilin Chen, Xiaoxia Ye, Chang Shu, Chunzhen Ren, Xuehan Wang, Hugang Jiang, Xiaodong Zhi, Linchan Li, Qianrong Li, Kai Liu, Xinke Zhao, Yingdong Li

**Affiliations:** 1 KweiChow Moutai Hospital, Renhuai, China; 2 School of Traditional Chinese and Western Medicine, Gansu University of Chinese Medicine, Lanzhou, China; 3 Gansu Province Key Laboratory of Chinese Medicine for the Prevention and Treatment of Chronic Diseases, Lanzhou, China; 4 Key Specialized Cardiovascular Laboratory, Key Clinical Specialty of the National Health Commission of the People’s Republic of China, National Administration of Traditional Chinese Medicine, Lanzhou, China; 5 The First Clinical Medical College, Lanzhou University, Lanzhou, Gansu China; 6 Gansu Medical College, Pingliang, China

**Keywords:** Bibliometrics, CiteSpace, VOSviewer, Visual Analysis, Myocardial Fibrosis, Glycolysis

## Abstract

**Background:**

Myocardial fibrosis represents a terminal pathological outcome in numerous cardiovascular diseases and is mechanistically linked to glycolytic dysregulation. However, systematic reviews integrating multidimensional evidence remain scarce.

**Objective:**

This study aims to conduct a visual analysis of myocardial fibrosis and glycolysis research using CiteSpace and VOSviewer, quantifying research trends and key contributors.

**Methods:**

Literature on “myocardial fibrosis” and “glycolysis” published between January 1, 2000, and December 31, 2024, was retrieved from the Web of Science, PubMed, and Scopus databases. After deduplication, 408 articles were included for analysis. CiteSpace and VOSviewer were employed to analyze data regarding countries, institutions, authors, and keywords.

**Results:**

A total of 408 studies were included. Annual publications on myocardial fibrosis and glycolysis exhibited a fluctuating upward trend, increasing from 1 (2000) to 51 (2024). These publications were divided into three phases: Initial Exploration (2000–2003, 1 article per year), Slow Fluctuation (2004–2009, 3–5 articles per year), and Rapid Development (2010–2024). During the Rapid Development phase, publications surged from 25 in 2019 to 44 in 2020, peaked in 2021–2022 (45 and 48 articles, respectively), and rebounded to 51 in 2024. Key contributors: The U.S. (177 studies, 32% of global output) and China (115 studies, 20%) were the leading contributors, collectively accounting for 52% of total publications; top institutions were Univ. of Alberta (15 Myocardial fibrosis representstudies) and Univ. of Utah (14 studies); core authors included Archer, S.L. (10 studies) and Chen, J.-X. (8 studies), with Lopaschuk, G.D. topping co-citations (117). High-frequency keywords: “heart failure” (107), “expression” (81), “metabolism” (60), “glycolysis” (44); high-centrality ones: “cardiac hypertrophy” (0.25), “fatty acid oxidation” (0.21). Emerging frontiers: “extracellular matrix” (burst intensity = 3.01, 2021–2024), “pulmonary hypertension” (3.61, 2022–2024). Clustering analysis identified 10 clusters (Silhouette Value = 0.7628, Modularity Q = 0.5292), including #0 pulmonary arterial hypertension (2014) and #10 mitochondrial dysfunction (2019).

**Conclusion:**

Our bibliometric analysis reveals three pivotal trends: (1) The U.S. (32%) and China (20%) lead international collaborative networks; (2) Glycolytic reprogramming, oxidative stress, and metabolic interventions are prevalent research focuses; (3) Mitochondrial dysfunction and extracellular matrix remodeling are emerging frontiers. These findings clarify the glycolysis-fibrosis axis and provide a framework for targeted anti-fibrotic therapies.

## Graphical Abstract

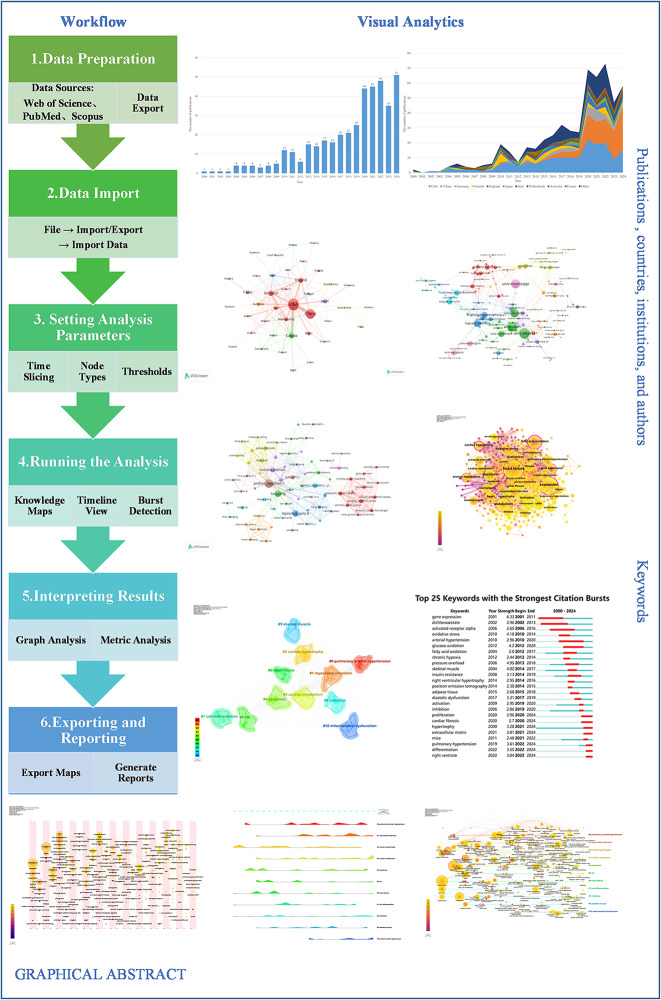

## Background

Cancer remains a major global public health challenge due to its high incidence and mortality. A key feature of cancer is the metabolic reprogramming of tumor cells, especially the abnormal activation of glycolysis, a phenomenon known as the “Warburg effect.” In this state, tumor cells depend on glycolysis for energy production even in the presence of adequate oxygen, which supports their unchecked proliferation and metastasis. This metabolic shift not only drives cancer progression but also offers promising therapeutic targets [1]. For example, inhibiting key glycolytic enzymes such as hexokinase 2 and lactate dehydrogenase A can effectively suppress tumor growth [2].

Chemotherapy remains a cornerstone of conventional cancer treatment. It primarily functions by disrupting the proliferation of rapidly dividing cells through mechanisms including DNA damage, inhibition of nucleic acid synthesis, and interference with cell cycle progression [3, 4]. Despite its widespread use, traditional chemotherapy faces significant limitations, including severe systemic toxicity to normal tissues (e.g., bone marrow suppression, gastrointestinal disorders, and neurotoxicity) and the inevitable development of drug resistance, which compromises long-term therapeutic efficacy [5]. To address these challenges, researchers have explored novel strategies to enhance chemotherapy outcomes, with a growing focus on integrating metabolic regulation into treatment regimens. Advances in nanocarrier technology, for instance, have enabled targeted delivery of chemotherapeutic agents, improving drug accumulation at tumor sites while reducing off-target effects [4, 5]. Furthermore, the development of synergistic combinations of chemotherapeutic agents with metabolic modulators or novel compounds (e.g., metal complexes with anticancer activity) has shown potential in overcoming drug resistance and enhancing cytotoxicity [3, 6]. Such progress in chemotherapy research not only provides insights into optimizing cancer treatment but also offers a framework for exploring metabolic dysregulation in other diseases, including cardiovascular disorders like myocardial fibrosis.

Recent studies have demonstrated that the efficacy of certain chemotherapeutic agents is closely linked to the metabolic regulation of tumor cells. By disrupting glycolysis in tumor cells, these drugs can increase cancer cell death and help overcome resistance [7, 8]. This strategy, known as “metabolic intervention,” not only offers new approaches to optimize chemotherapy but also provides insights into metabolic abnormalities in other diseases. For example, in myocardial fibrosis, abnormal activation of glycolysis promotes fibroblast proliferation and excessive extracellular matrix deposition, both of which are crucial for disease progression. Key mechanisms linking glycolysis to fibrosis include: (1) Hypoxia-inducible factor-1α (HIF-1α) activation driving the expression of glycolytic enzymes and facilitating fibroblast-to-myofibroblast differentiation via pathways like TGF-β; (2) metabolic competition between cell types under stress, favoring glycolytic fibroblasts; and (3) lactate accumulation promoting extracellular matrix remodeling and pro-fibrotic signaling through its effects on histone lactylation and receptors like GPR81 [9–13].

Notably, cancer and myocardial fibrosis share similar regulatory mechanisms governing glycolysis. For instance, activation of HIF-1α upregulates glycolytic enzyme expression in both diseases. Additionally, lactate, a glycolytic byproduct, contributes to remodeling the tumor microenvironment and, through epigenetic modifications such as histone lactylation, promotes myocardial fibrosis progression [9]. Therefore, investigating the regulation of glycolysis in myocardial fibrosis can help identify specific therapeutic targets and deepen our understanding of metabolic disorders, underlining the wider significance of this research.

Myocardial fibrosis is a common pathological outcome in the final stages of various cardiovascular diseases, including hypertension, myocardial infarction, and diabetic cardiomyopathy. This condition is marked by the abnormal activation of cardiac fibroblasts, excessive extracellular matrix (ECM) deposition, and increased myocardial stiffness. These changes result in impaired diastolic function, arrhythmias, and potentially lead to heart failure [14]. Although treatments such as renin–angiotensin system inhibitors and anti-inflammatory therapies have shown some progress, their clinical efficacy remains limited, and the long-term prognosis for patients remains poor [15]. Recent research has demonstrated a strong link between myocardial fibrosis and metabolic disturbances, particularly the abnormal activation of glycolysis. This process could serve as a therapeutic target through regulating fibroblast phenotype changes, inflammatory responses, and oxidative stress [16].

Mature cardiomyocytes predominantly utilize fatty acid oxidation (FAO) for energy production, while fibroblasts rely on glycolysis to fuel proliferation and ECM secretion [17]. Under pathological conditions (e.g., ischemia, hypoxia, inflammation), however, metabolic reprogramming occurs in both cardiomyocytes and fibroblasts. Mitochondrial dysfunction drives cardiomyocytes toward glycolysis, whereas fibroblasts amplify glycolytic flux. This metabolic competition exacerbates fibrotic progression [18]. HIF-1α facilitates the differentiation of fibroblasts into myofibroblasts by upregulating key glycolytic enzymes, such as HK2 and LDHA, and stimulates collagen synthesis through the TGF-β/Smad3 pathway [10]. Lactate, a byproduct of glycolysis, not only regulates ECM remodeling but also promotes pro-fibrotic signaling by activating the G protein-coupled receptor 81 (GPR81) [11]. These findings suggest that spatiotemporal modulation of glycolytic metabolism could offer a novel approach for reversing myocardial fibrosis.

Glycolysis is a key pathway in cellular energy metabolism that not only produces ATP but also plays a role in cell fate determination and epigenetic regulation. In myocardial fibrosis, the abnormal activation of glycolysis has a “double-edged sword” effect. On one hand, fibroblasts increase glycolysis through the “Warburg effect,” rapidly generating ATP and precursors (such as NADPH and amino acids) to support proliferation, migration, and collagen secretion [10, 12]. Lactate accumulation directly stabilizes HIF-1α by inhibiting prolyl hydroxylases, activates the NLRP3 inflammasome, and promotes histone lactylation, which together amplify pro-fibrotic signaling [13]. Conversely, moderate inhibition of glycolysis may slow fibrosis progression by restoring the balance of oxidative phosphorylation, reducing oxidative stress, or inducing autophagy. For example, metformin inhibits glycolysis by activating the AMPK pathway, which reduces collagen deposition after myocardial infarction [19, 20]. SGLT2 inhibitors (e.g., empagliflozin) downregulate HK2 expression, improving fibrosis in diabetic cardiomyopathy [21].

However, the spatiotemporal variability in glycolysis regulation, its cell type specificity, and its interactions with classical fibrosis pathways, such as TGF-β and Wnt/β-catenin, are not yet fully understood. Therefore, integrating multidimensional evidence to clarify the causal link between metabolic reprogramming and fibrosis is essential for developing targeted therapies. Although existing studies have started to explore the role of glycolysis in myocardial fibrosis, the field’s knowledge structure, evolving research trends, and interdisciplinary collaboration models have not been systematically analyzed. Traditional reviews often rely on qualitative summaries, which makes it difficult to quantify trends or identify innovative directions in the field. Bibliometrics, which applies mathematical and statistical methods to analyze publication data, addresses these limitations. Visualization tools such as CiteSpace and VOSviewer are particularly well-suited for this purpose, enabling the mapping of scientific landscapes to identify key contributors (authors, institutions, countries), collaboration networks, research hotspots, and emerging trends through co-citation, co-authorship, and co-occurrence analysis. This approach maps the field by identifying key researchers, influential institutions, and international collaboration patterns, thereby fostering resource integration and cross-border cooperation [22]. Timeline analysis tracks the shifting focus of “glycolysis-fibrosis” research, while burst detection identifies rapid increases in key terms, offering early indicators of future breakthroughs. This information will offer valuable insights to future researchers and enhance understanding of the current state of the field.

## Methods

**Fig. 1:**
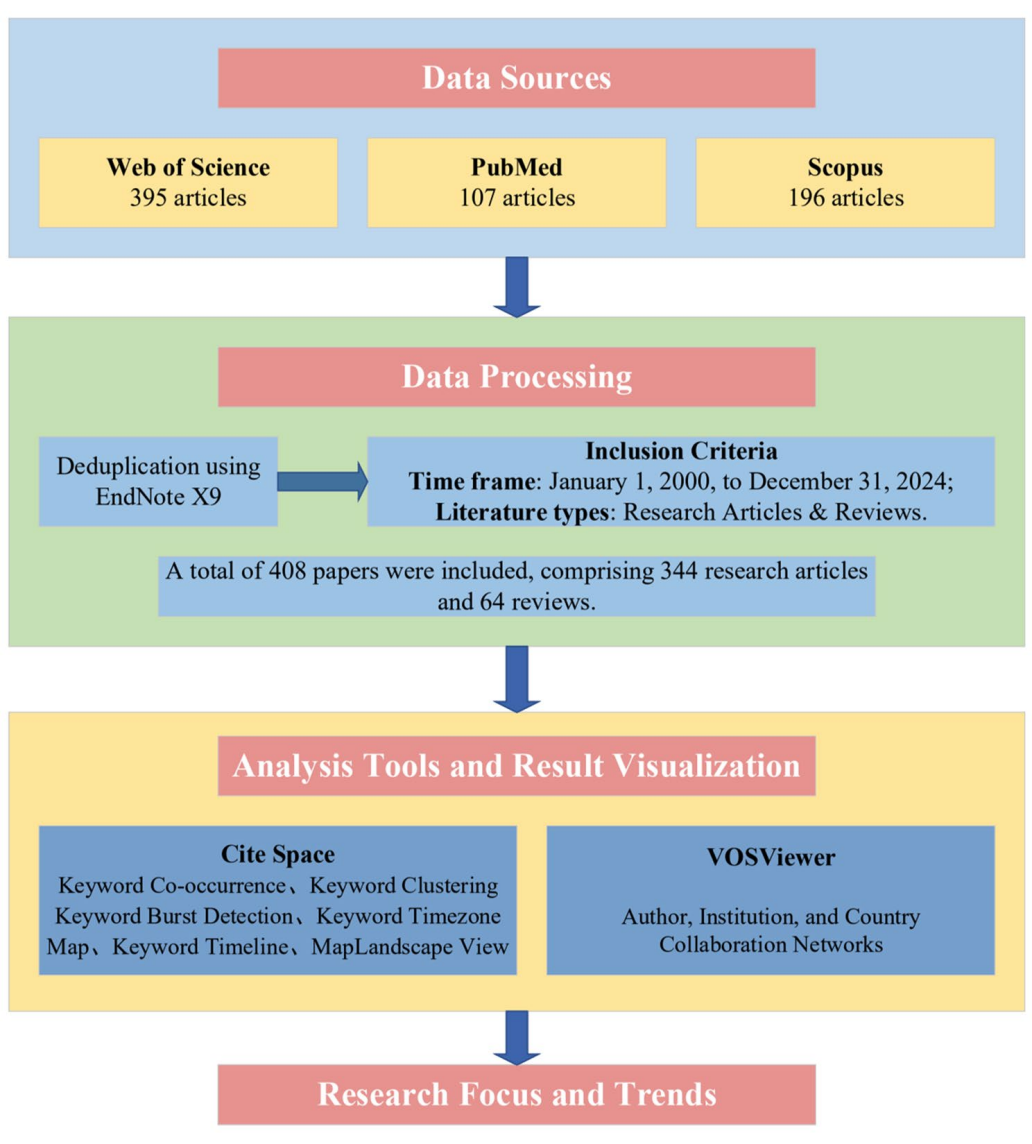
The research framework and workflow of the bibliometric analysis on myocardial fibrosis and glycolysis

The overall research framework of this bibliometric analysis is summarized in Fig. 1. Briefly, data were retrieved from three major databases, processed according to predefined criteria, and analyzed using CiteSpace and VOSviewer to identify research foci and trends.

### Data sources and search strategy

Database Selection: This analysis utilized three major international databases: Web of Science (WOS) Core Collection, PubMed, and Scopus. This combination is recognized as a standard and robust approach in bibliometrics, providing extensive coverage of high-impact, peer-reviewed literature across the life sciences and medicine. While other databases such as Embase offer valuable coverage, its content in the biomedical field has substantial overlap with Scopus. The tri-database combination of WOS, Scopus, and PubMed has been methodologically shown to capture the vast majority of significant scientific output, thereby ensuring comprehensiveness while maintaining data homogeneity for software processing.

Exclusion of Regional Databases: Regional databases such as China National Knowledge Infrastructure (CNKI) were not included. This decision was primarily based on the technical requirements of the bibliometric software (CiteSpace and VOSviewer), which are optimized for the specific data structure and export formats of international databases. Integrating CNKI, which has a different and incompatible data schema, would introduce significant technical artifacts and invalidate network analyses. Furthermore, the core research from leading Chinese institutions in this field is predominantly published in international English-language journals indexed by our selected databases, as evidenced by China’s prominent role in our results.

Keyword Search: We systematically queried Web of Science, PubMed, and Scopus using predefined search strings for myocardial fibrosis and glycolysis. Keywords for myocardial fibrosis included “myocardial fibrosis,” “myocardial fibrotic,” “myocardial fibrosis,” “heart fibrosis,” “myocardial interstitial fibrosis,” “cardiac remodeling,” “ventricular remodeling,” “fibrotic cardiomyopathy,” and “myocardial scarring.” Keywords for glycolysis included “glycolysis,” “glycolytic,” “Embden-Meyerhof pathway,” “aerobic glycolysis,” “anaerobic glycolysis,” and “Warburg effect.”

Search Strategy: The search queries varied across databases. In WOS, the query was: TS=((“myocardial fibrosis” OR “myocardial fibrotic” OR “myocardial fibrosis” OR “heart fibrosis” OR “myocardial interstitial fibrosis” OR “cardiac remodeling” OR “ventricular remodeling” OR “fibrotic cardiomyopathy” OR “myocardial scarring”) AND (“Glycolysis” OR “Glycolytic” OR “Embden-Meyerhof pathway” OR “aerobic glycolysis” OR “anaerobic glycolysis” OR “Warburg effect”)) NOT TS=(cancer OR tumor OR oncology). In PubMed, the query used was: ((“myocardial fibrosis“[Title/Abstract] OR “myocardial fibrotic“[Title/Abstract] OR “myocardial fibrosis“[Title/Abstract] OR “heart fibrosis“[Title/Abstract] OR “myocardial interstitial fibrosis“[Title/Abstract] OR “cardiac remodeling“[Title/Abstract] OR “ventricular remodeling“[Title/Abstract] OR “fibrotic cardiomyopathy“[Title/Abstract] OR “myocardial scarring“[Title/Abstract]) AND (“Glycolysis“[MeSH] OR glycolys*[Title/Abstract] OR “Embden-Meyerhof pathway“[Title/Abstract] OR “aerobic glycolysis“[Title/Abstract] OR “anaerobic glycolysis“[Title/Abstract] OR “Warburg effect“[Title/Abstract])). In Scopus, the query used was: TITLE-ABS-KEY((“myocardial fibrosis” OR “myocardial fibrotic” OR “myocardial fibrosis” OR “heart fibrosis” OR “myocardial interstitial fibrosis” OR “cardiac remodeling” OR “ventricular remodeling” OR “fibrotic cardiomyopathy” OR “myocardial scarring”) AND (glycolys* OR “Embden-Meyerhof pathway” OR “aerobic glycolysis” OR “anaerobic glycolysis” OR “Warburg effect”)).

### Search limitations

The search encompassed literature published from January 1, 2000, to December 31, 2024. Only research articles, reviews, and clinical trials in English were included. Studies with the terms “cancer,” “tumor,” or “oncology” in the title or abstract were excluded.

In addition to the language restriction (English only) and exclusion of cancer-related articles, the following inclusion and exclusion criteria were applied: (1) only research articles, reviews, and clinical trials were included; conference abstracts, preprints, and other gray literature were excluded; (2) both animal and human studies were eligible for inclusion, with no restriction on study type; (3) duplicate records were identified and removed using EndNote X9 based on matching titles, authors, and publication years.

**Fig. 2:**
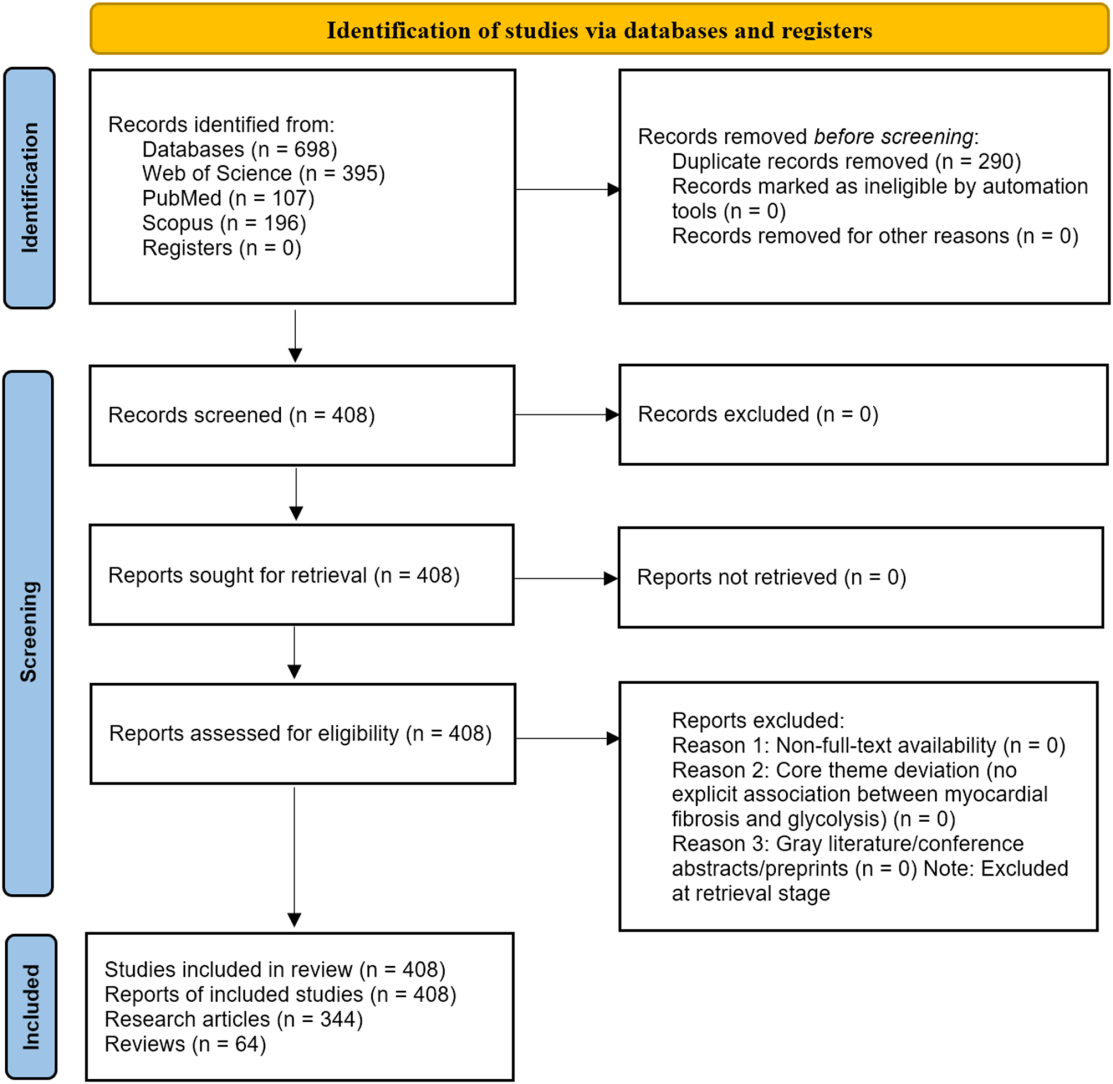
PRISMA-style flow diagram of literature screening and inclusion for myocardial fibrosis and glycolysis research 2000–2024

To clarify the literature screening workflow, a PRISMA 2020-style flow diagram (Fig. 2) was constructed to visualize each step. Two independent reviewers conducted title/abstract and full-text screening; any discrepancies were resolved via discussion with a third reviewer. Notably, gray literature (e.g., technical reports, government documents), conference abstracts, and preprint articles were excluded at the database retrieval stage (not included in the initial 698 records) due to potential limitations in peer review and data completeness, as outlined in our search strategy.

In the literature screening and deduplication process, the initial search yielded 395 articles from Web of Science (WOS), 107 from PubMed, and 196 from Scopus. After deduplication with EndNote X9, 408 papers were included, consisting of 344 research articles and 64 reviews, as shown in Fig. 2.

### Visualization analysis tools

This study employed CiteSpace (version 6.2.R4) and VOSviewer (version 1.6.18) for bibliometric co-occurrence analysis and visualization creation. The analysis results were imported into Microsoft Excel 2019 for further charting and visualization. In the visual maps, nodes represent various research elements such as authors, institutions, keywords, and papers, while the connecting lines illustrate co-occurrence and co-citation relationships between them. The number and size of nodes are proportional to their importance or frequency in the research field, while the thickness of the lines indicates the strength of relationships between the nodes. To address the temporal bias introduced by raw citation counts (which tend to favor older publications), we supplemented our analysis with the Citation Per Paper (CPP) metric—defined as the total citations of a given entity (e.g., country or institution) divided by its number of publications. This provides a more normalized measure of impact. In further analysis, centrality is a key metric for assessing the importance of a node within the network. When a node’s centrality value exceeds 0.1, it indicates that the node holds a central position in the network and is regarded as a key node in the field. These visualization tools enable the identification of dominant authors, key institutions, major journals, and their interrelationships, aiding in the identification of trends and key issues in the field’s development.

Expert Validation of Keyword Clustering: To enhance the thematic accuracy and interpretability of the keyword clusters generated by CiteSpace, we conducted a post-hoc expert validation process. Two independent cardiovascular metabolism researchers with over five years of experience in the field reviewed the keyword composition and cluster labels (e.g., #0 pulmonary arterial hypertension, #10 mitochondrial dysfunction). They assessed whether the clusters meaningfully represented distinct and biologically plausible research themes. Discrepancies were resolved through discussion. This expert validation confirmed that the clusters accurately reflected established and emerging research directions, thereby strengthening the interpretative validity of our bibliometric findings.

### Validation of search strategy

To ensure the comprehensiveness and accuracy of our search strategy, we employed a multi-step validation process. First, the initial search strings were developed through an iterative process, including consultation with a specialist librarian and preliminary testing to refine keyword combinations. Second, we cross-verified the retrieved records against a set of known seminal publications in the field (e.g., Stanley et al., 2005; Travers et al., 2016) to confirm that our strategy captured key studies. Finally, the final search syntax was independently reviewed by two authors to minimize the risk of omission or misclassification. These steps collectively enhance the reliability and reproducibility of our literature retrieval process.

### Note on data presentation

All figures in this study are derived from bibliometric analyses (e.g., publication counts, collaboration networks, keyword co-occurrence) and do not represent experimental measurements. As such, error bars are not applicable, as the data are based on discrete counts and network metrics rather than experimental replicates.

### Statistical analysis methods

Table 1 illustrates the statistical tests selected to assess the significance of assertions regarding “dominance,” “emerging trends,” and “centrality shifts.” These tests were tailored to three data types: count data (publication/keyword frequency), network data (centrality), and time-series data (annual trends). All analyses were performed using SPSS 26.0, R 4.3.0 (packages: segmented/igraph), and the built-in statistical module of CiteSpace 6.2.R4, where the significance level was set at α = 0.05.

**Table 1:** Statistical methods and corresponding test purposes for key claims in the bibliometric study of myocardial fibrosis and glycolysis

Claim Type	Data Characteristics	Statistical Method	Purpose of Test
Dominance of countries/institutions/authors	Publication counts (non-normal)	Kruskal-Wallis H test + Dunn’s post-hoc test (Bonferroni-corrected)	Compare publication differences among Top 10 entities to verify if “dominant entities” differ significantly from others
Centrality of countries/keywords	Network centrality values (normal approximation)	Z-test (Z = (Observed value - Mean)/Standard deviation)	Verify if core nodes (countries/keywords) have centrality significantly higher than the network average (Z > 1.96 indicates *p* < 0.05)
Annual publication trends	Time-series data	Segmented regression	Test for significant differences in annual growth rates across the three phases (“Initial Exploration–Slow Fluctuation–Rapid Development”)
Validity of clustering structure	Network clustering indices (Modularity Q/Silhouette Value)	Permutation test (*n* = 1000)	Verify if Modularity Q is significantly higher than that of randomly generated networks to rule out random clustering

## Results

### Annual publication volume statistics and analysis

**Fig. 3:**
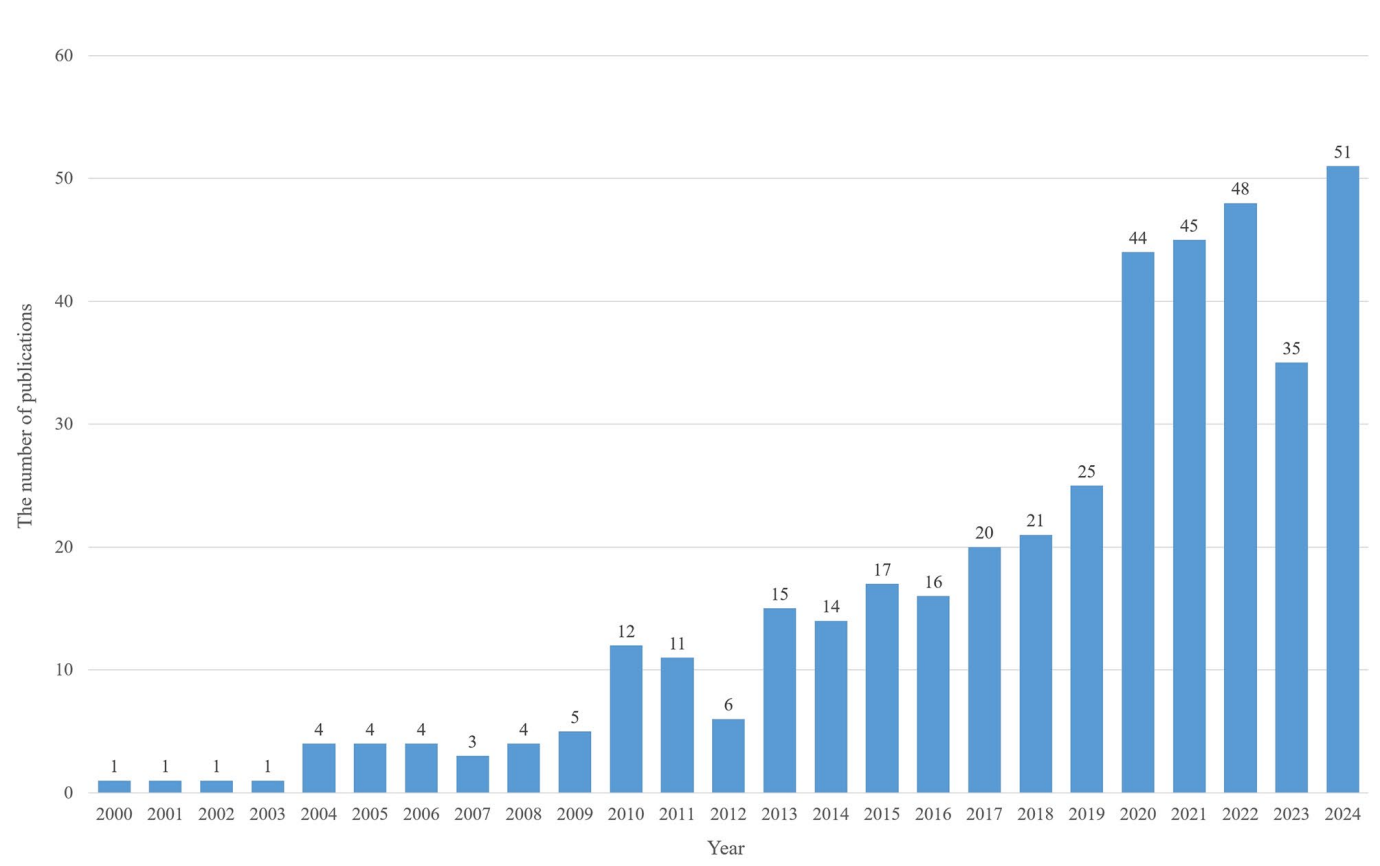
Global annual publication trends in the field of “glycolysis and myocardial fibrosis” from 2000 to 2024 (Data source: Web of Science, Scopus, PubMed)

This study employed bibliometric methods to analyze the annual publication trends and contributions from different countries/regions in the field of “myocardial fibrosis and glycolysis” between 2000 and 2024 (Figs. 2 and 3). The data indicate that research in this field has progressed dynamically, from slow accumulation to rapid growth, with notable differences in research output across countries.

As shown in Fig. 3, the global annual publication volume increased from 1 article in 2000 to 51 in 2024 (data as of December 31, 2024), showing a fluctuating upward trend. Segmented regression analysis of annual publications against time identified two significant breakpoints (2003 and 2009, breakpoint test *p* < 0.001): Initial Exploration (2000–2003): Annual growth rate *β* = 0.02 (95% CI: -0.05–0.09, *p* = 0.58; no significant growth); Slow Fluctuation (2004–2009): Annual growth rate *β* = 0.45 (95% CI: 0.08–0.82, *p* = 0.03; moderate significant growth); Rapid Development (2010–2024): Annual growth rate *β* = 3.21 (95% CI: 2.87–3.55, *p* < 0.001; highly significant and rapid growth); Conclusion: Statistically significant differences existed in growth rates across the three phases (interaction term F = 47.2, df = 2, *p* < 0.001), confirming the “Rapid Development Phase” as a valid trend.

Initial Exploration Phase (2000–2003): The annual publication volume remained low, with only one article per year, indicating that the research was in its early stages with limited exploration and no large-scale studies. Slow Fluctuation Phase (2004–2009): The publication volume remained stable at 4 articles from 2004 to 2006, dropped slightly to 3 in 2007, increased back to 4 in 2008, and rose to 5 in 2009. This phase showed small fluctuations, indicating a gradual increase in research attention, though it remained in the accumulation phase. Rapid Development Phase (2010–2024): In 2010, the publication volume surged to 12 articles, slightly decreased to 11 in 2011, then fluctuated between 6 and 17 from 2012 to 2016. From 2017 to 2019, it rose from 20 to 25 articles. In 2020, it increased explosively to 44, peaking at 45 publications in 2021 and 48 in 2022. Following a transient decline to 35 in 2023, publications rebounded to 51 in 2024. This phase saw a significant increase in publication volume, particularly in 2020, when related articles surged from 25 to 44, driven by several factors. The COVID-19 pandemic heightened academic interest in the association between viral infections, myocardial injury, and glycolytic metabolism. This was particularly evident in studies exploring cytokine storms and the mechanisms of myocardial fibrosis in COVID-19 patients. Meanwhile, advances in technologies like metabolomics and single-cell sequencing provided new methods to explore glycolytic reprogramming, accelerating research in the field. Additionally, the release of specialized guidelines by the American Heart Association (AHA), combined with favorable funding policies from various countries, significantly increased investment and focus on related topics. Review articles in authoritative journals like Circulation Research systematically reviewed the relationship between glycolysis and fibrosis, accelerating the dissemination and application of research findings. Furthermore, the use of bibliometric tools allowed researchers to accurately identify and explore emerging research hotspots, such as “glycolytic reprogramming.”

Analyzing highly cited papers from 2020 reveals that most focused on the intersection of glycolysis and inflammation/oxidative stress mechanisms. These studies align with the “cytokine storm” phenomenon in COVID-19, further validating the impact of multi-factorial interactions in this field. In summary, these factors have collectively accelerated the rapid development of this research area, making it a focal point in the academic community and attracting substantial research involvement. The depth and breadth of research continue to grow.

Overall, the publication volume shifted from sporadic exploration in the early stages to high-frequency output in later years, reflecting the transformation of research on “myocardial fibrosis and glycolysis” from a niche topic into an academic hotspot. This trend suggests the field will continue to expand, leading to more innovative outcomes in the future.

**Fig. 4:**
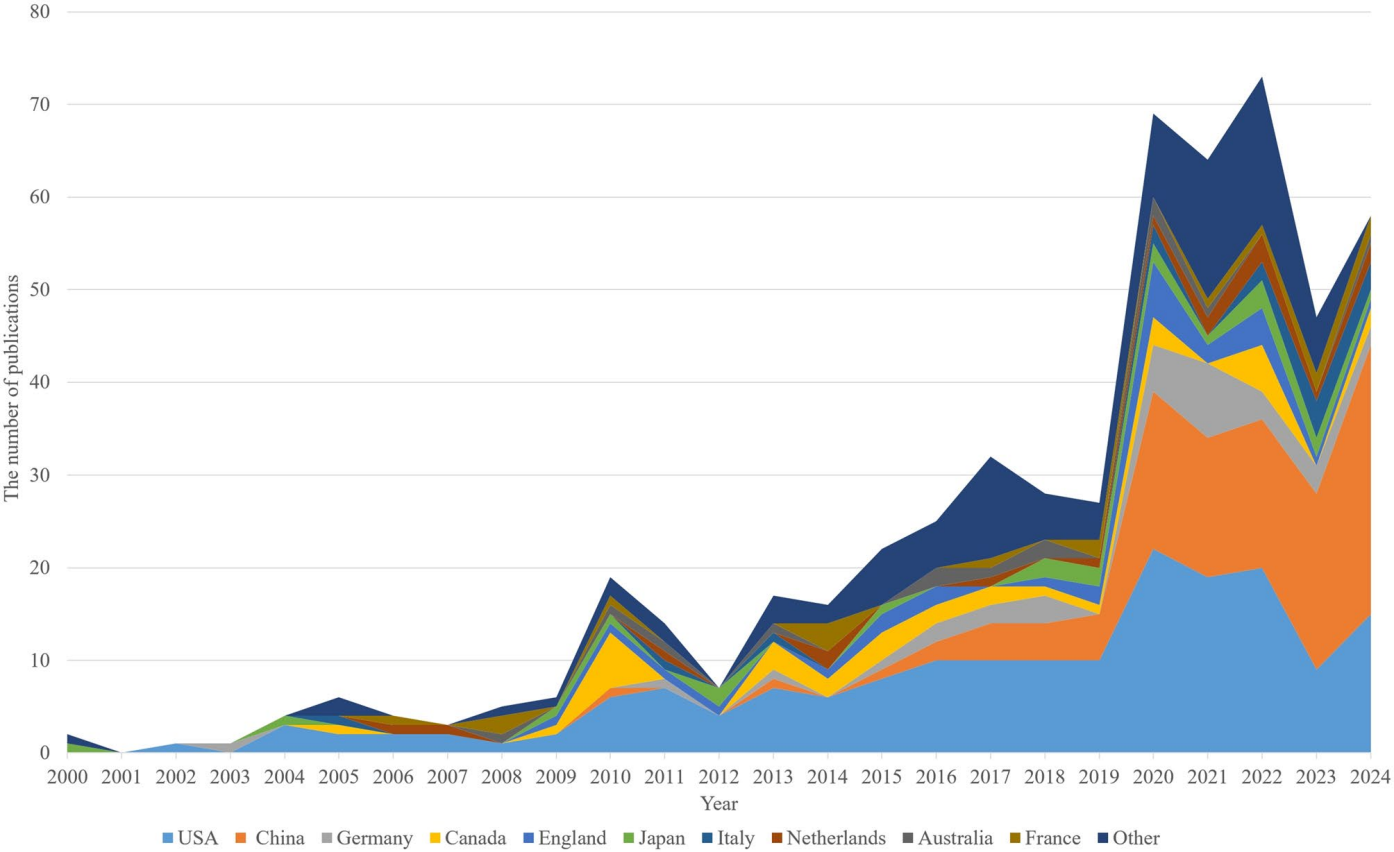
Distribution of cumulative publication volume by major countries

The United States (177 publications, 32%) and China (115 publications, 20%) emerged as the primary contributors (Fig. 4), accounting for 52% of global publications. Kruskal-Wallis H test for publication counts of the top 10 countries showed overall significant differences (H = 126.8, df = 9, *p* < 0.001). Dunn’s post-hoc test (Bonferroni-corrected) revealed: The U.S. had significantly more publications than all countries except China (*p* < 0.001); China had significantly more publications than Germany (32 articles), Canada (33 articles), and other countries (*p* < 0.01); The combined contribution of the U.S. and China (52%) was significantly higher than the average contribution of the remaining 8 countries in the top 10 (4.8%) (Z = 8.92, *p* < 0.001); The United States consistently maintained a high publication volume, with a significant contribution to the overall growth in the later stages of research. China has shown a significant upward trend in publications, emerging as a key driver of the field’s development. Countries such as Germany, Canada, and England have also continued to contribute research, with several nations advancing studies on myocardial fibrosis and glycolysis. Changes in annual publication volume reflect not only growing research interest but also increasing global attention to the exploration of mechanisms and therapeutic strategies, providing a solid foundation for future research.

### Bibliometric analysis of countries, institutions, and authors

**Fig. 5:**
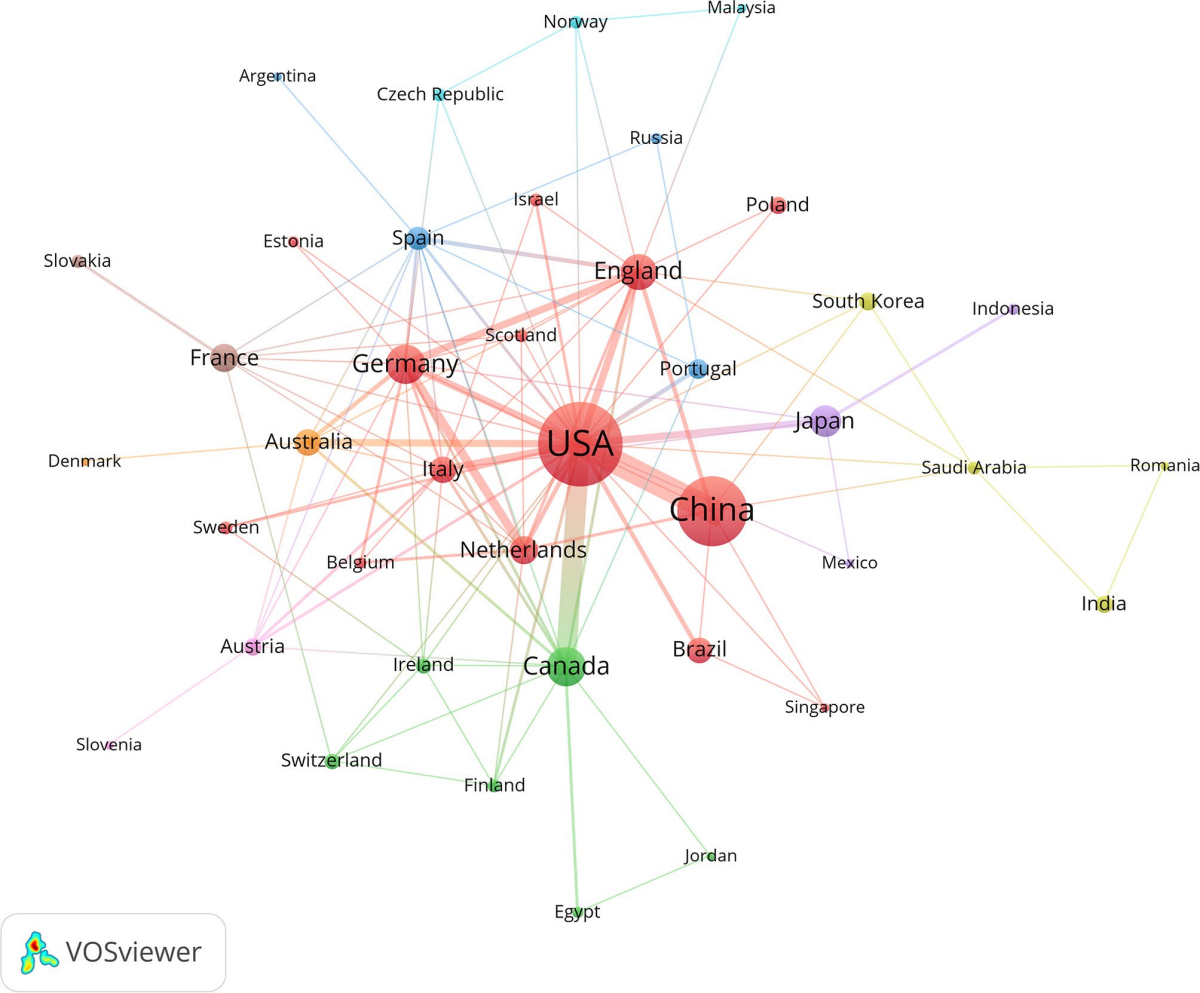
Country collaboration network map (node size = publication volume, line thickness = collaboration frequency, color = clustering). Only 32 countries with publication volumes ≥ 2 are displayed. Ten distinct collaboration clusters, represented by different colors, have formed, with a total connection strength of 188

**Fig. 6:**
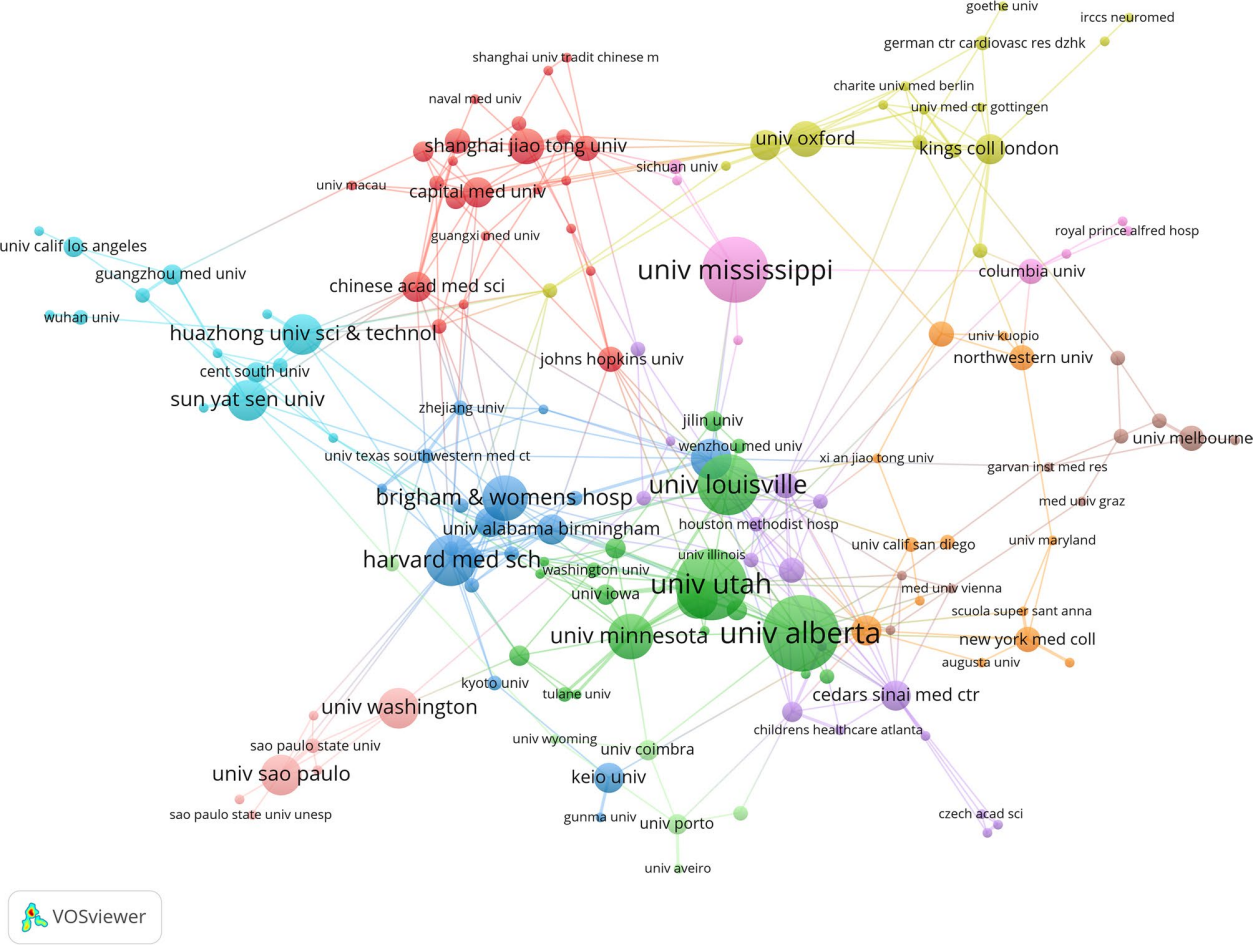
Institutional collaboration network map (node size = publication volume, line thickness = collaboration frequency, color = clustering). A total of 734 institutions are represented, with 175 institutions shown, having a publication volume ≥ 2. The map displays 15 distinct collaboration clusters, represented by different colors, with a total connection strength of 464

**Fig. 7:**
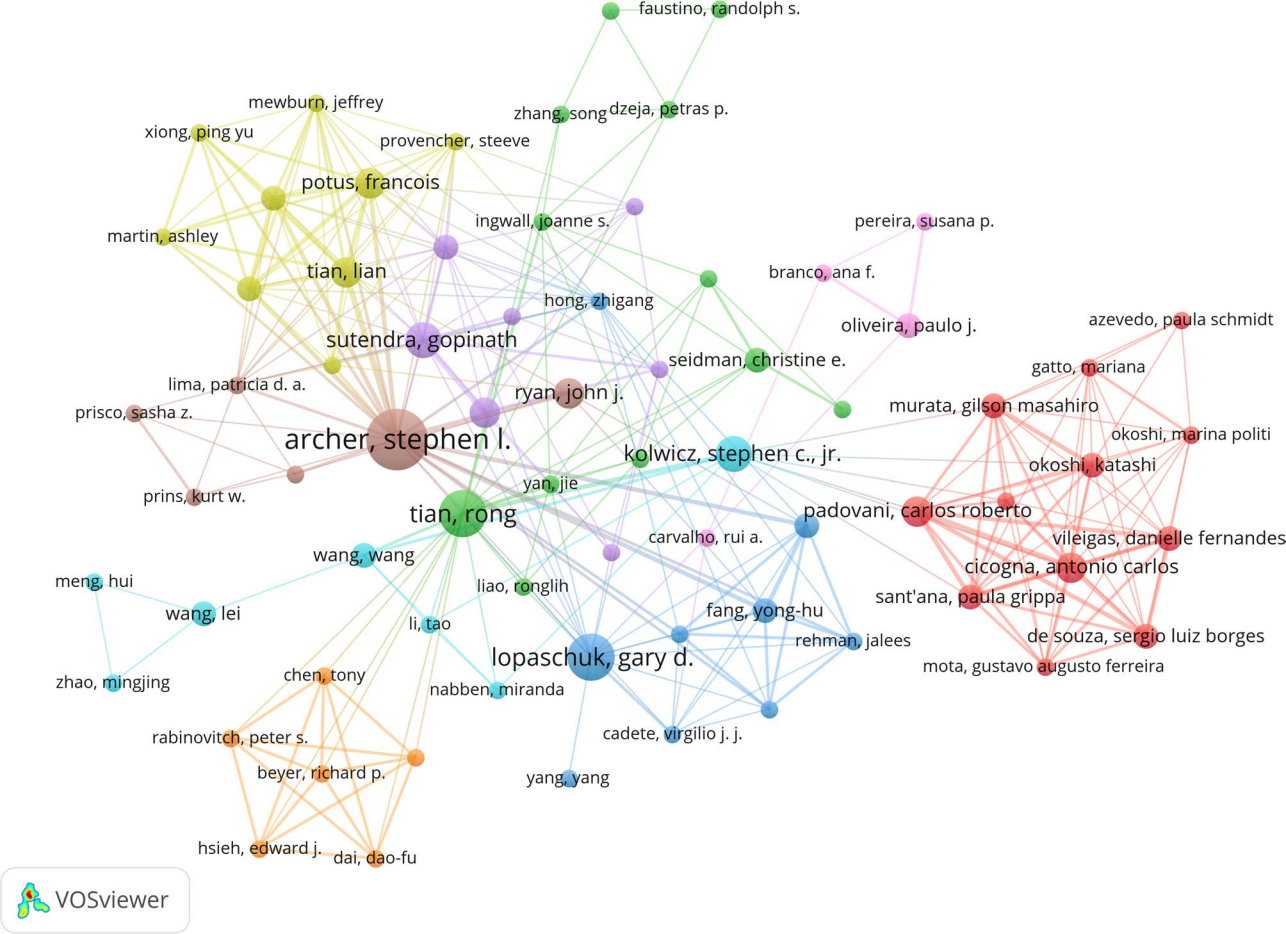
Author collaboration network map (node size = publication volume, line thickness = collaboration frequency, color = clustering), showing 290 authors with a publication volume of 2 or more. Nine distinct collaboration clusters are formed, represented by different colors, with a total connection strength of 428

This study used VOSviewer to visually analyze the collaboration networks of countries, institutions, and authors (Figs. 5, 6 and 7), along with publication and citation data (Tables 2, 3, 4 and 5). This approach systematically reveals global collaboration patterns and the distribution of key research forces in the field of “glycolysis and myocardial fibrosis.” The results indicate that international collaboration follows a “multi-center, regionally clustered” model, with leading institutions and research teams playing a dominant role.

Figure 5; Table 2 reveal that research on myocardial fibrosis is mainly concentrated in the United States and China, with both countries holding central positions in the global collaboration network. The betweenness centrality of nodes in the national collaboration network was calculated, with a network average of 0.03 (SD = 0.06). Z-test results showed that the U.S. had a betweenness centrality of 0.28 (Z = 4.23, *p* < 0.001), while China had a betweenness centrality of 0.19 (Z = 3.17, *p* < 0.001); Non-core countries (e.g., Canada: centrality = 0.08, Z = 0.83, *p* = 0.41; Germany: centrality = 0.07, Z = 0.67, *p* = 0.50) showed no statistical significance in centrality; The U.S. and China are statistically significant core nodes in the collaboration network, while other countries lack significant core status. The United States leads with 177 publications, 11,950 citations, and 28 collaboration links, underscoring its leadership in global research. China follows closely, with 115 publications and 5,100 citations, emphasizing its growing academic influence in the field, despite having a slightly lower publication count than the United States. Other prominent countries include Canada, Germany, the United Kingdom, and Japan. Canada and Germany, with 33 and 32 publications, rank third and fourth, reflecting their strong research capabilities in the field and their key roles in international collaboration. Notably, countries with fewer publications, such as Japan and the Netherlands, still exert considerable influence in the collaboration network, especially in partnerships with the United States and China, highlighting the essential role of international cooperation in advancing research.

As shown in Fig. 6; Table 3, several top institutions have made significant contributions to myocardial fibrosis research. The University of Alberta ranks first, with 15 publications and 3,436 citations. The University of Utah and University of Mississippi follow closely, ranked second and third, with 14 and 13 publications, respectively. Kruskal-Wallis H test for publication counts of the top 10 institutions showed significant differences (H = 45.2, df = 9, *p* < 0.001). Dunn’s post-hoc test revealed: The University of Alberta (15 articles) had significantly more publications than all top 10 institutions except the University of Utah (14 articles) (*p* < 0.01); No significant difference existed between the University of Utah and the University of Mississippi (13 articles) (*p* = 0.62), but both had significantly more publications than Harvard Medical School (10 articles, *p* < 0.05); The University of Alberta is the statistically significant most productive institution, with hierarchical differences in contributions among the top 3 institutions. These institutions have not only high publication output but also a significant citation frequency, highlighting the impact of their research on the field. Additionally, U.S. institutions, such as Harvard Medical School and Brigham and Women’s Hospital, have made significant academic contributions. Although their publication counts are lower, their academic influence and inter-institutional collaboration are notable. The research from these institutions has advanced basic studies on myocardial fibrosis and facilitated the exploration of clinical applications and new treatment strategies.

As shown in Fig. 7; Table 4, research on myocardial fibrosis is led by several prominent scholars. Stephen L. Archer leads in publication volume, citation count, and TLS, highlighting his significant research impact and central role in academic collaboration. Gopinath Sutendra, despite having fewer publications, has a high citation count, suggesting that his work is of high quality. Hai-Feng Zhang and Steven P. Jones have extensive academic collaborations, but their relatively lower citation counts may indicate their significant roles in academic exchanges. G.D. Lopaschuk ranks first with 117 total citations, underscoring the foundational nature of his research in this field. Overall, Stephen L. Archer is the central figure in this field, while others, such as Gopinath Sutendra and Hai-Feng Zhang, may represent emerging scholars with potential and key nodes in the academic collaboration network.

Table 5 presents the five most highly cited papers in the field of myocardial fibrosis and glycolysis, highlighting foundational research and key areas of interest. Three papers examine the mechanisms underlying myocardial energy metabolism (#1, #3, #5). The other two focus on clinical conditions and therapies, specifically pulmonary hypertension (#2) and the use of SGLT2 inhibitors in heart failure (#4). Stanley et al.‘s (2005) review is the most influential work, with 1,545 citations. This paper provides a comprehensive overview of myocardial substrate metabolism in heart failure. More recent publications from 2018 to 2019 (#4, #5) indicate increasing interest in metabolic interventions for heart failure treatment. All these papers appeared in top journals, including Physiological Reviews, Nature Reviews Cardiology, and JACC, demonstrating the high quality and recognition of research in this area.

**Table 2:** Top 10 countries by publication volume

Rank	Country	Documents	Citations	CPP	Links	TLS	Z-Score (Centrality)	*p*-Value	Dominance Validation
1	USA	177	11,950	67.5	28	88	4.23	0.001	Yes
2	China	115	1940	16.9	8	27	3.17	0.001	Yes
3	Canada	33	5100	154.5	13	31	0.83	0.41	No
4	Germany	32	1301	40.7	16	35	0.67	0.50	No
5	England	26	1018	39.2	16	30	1.00	0.32	No
6	Japan	20	848	42.4	5	10	0.50	0.62	No
7	Netherlands	16	501	31.3	10	20	0.83	0.41	No
8	France	16	347	21.7	9	10	0.67	0.50	No
9	Italy	14	1970	140.7	14	21	1.00	0.32	No
10	Australia	14	592	42.3	8	15	0.67	0.50	No

Note: CPP (Citations Per Paper) = Total Citations / Documents

**Table 3:** Top 10 high-output institutions

Rank	Organization	Documents	Citations	CPP	Links	TLS
1	University of Alberta	15	3436	229.1	18	18
2	University of Utah	14	1782	127.3	17	25
3	University of Mississippi	13	774	59.5	7	7
4	University of Louisville	12	698	58.2	9	11
5	Harvard Medical School	10	257	25.7	18	25
6	Brigham and Women’s Hospital	9	588	65.3	16	24
7	University of Minnesota	9	977	108.6	13	19
8	Duke University	8	352	44.0	14	15
9	Huazhong University of Science and Technology	8	119	14.9	10	11
10	Queen’s University	8	1418	177.3	7	11

**Table 4:** The Top 10 most prolific authors and co-cited authors

Rank	Author	Documents		Citations	TLS	Rank	Author	Co-citations
1	Archer, Stephen L.		10	1873	50	1	Lopaschuk, G.D.	117
2	Chen, Jian-Xiong		8	303	21	2	Stanley, W.C.	82
3	Zeng, Heng		8	303	21	3	Piao, L.	65
4	Tian, Rong		7	743	24	4	Taegtmeyer, H.	64
5	Lopaschuk, Gary D.		7	733	18	5	Kolwicz, S.C.	62
6	Jones, Steven P.		6	450	33	6	Neubauer, S.	58
7	Hill, Bradford G.		6	437	26	7	Archer, S.L.	57
8	He, Xiaochen		6	225	19	8	Michelakis, E.D.	57
9	Zhang, Hai-Feng		5	105	34	9	Doenst, T.	56
10	Sutendra, Gopinath		5	726	23	10	Sutendra, G.	49

**Table 5:** Top 5 most cited papers

Rank	Paper Title	Journal	Citations	Year	Citations per Year
1	Myocardial substrate metabolism in the normal and failing heart	Physiological Reviews	1545	2005	81.3
2	Pulmonary arterial hypertension: pathogenesis and clinical management	The BMJ	652	2018	108.7
3	Amp-activated protein kinase mediates ischemic glucose uptake and prevents postischemic cardiac dysfunction, apoptosis, and injury	The Journal of Clinical Investigation	625	2004	31.3
4	Empagliflozin ameliorates adverse left ventricular remodeling in nondiabetic heart fallure by enhancing myocardial energetics	Journal of the American College of Cardiology	448	2019	89.6
5	Metabolic remodelling in heart failure	Nature Reviews Cardiology	429	2018	71.5

Note: Citations per Year = Total Citations / (2025 - Publication Year)

### Bibliometric analysis of keyword co-occurrence and clustering

Keyword co-occurrence occurs when different keywords appear together in the same document. Analyzing co-occurrence frequency and network relationships reveals the connections between research topics, identifies key hotspots, and uncovers potential knowledge structures. Keywords with high co-occurrence frequencies usually represent key research directions, while high centrality keywords act as “bridges” between topics, reflecting their role in cross-disciplinary research.

**Fig. 8:**
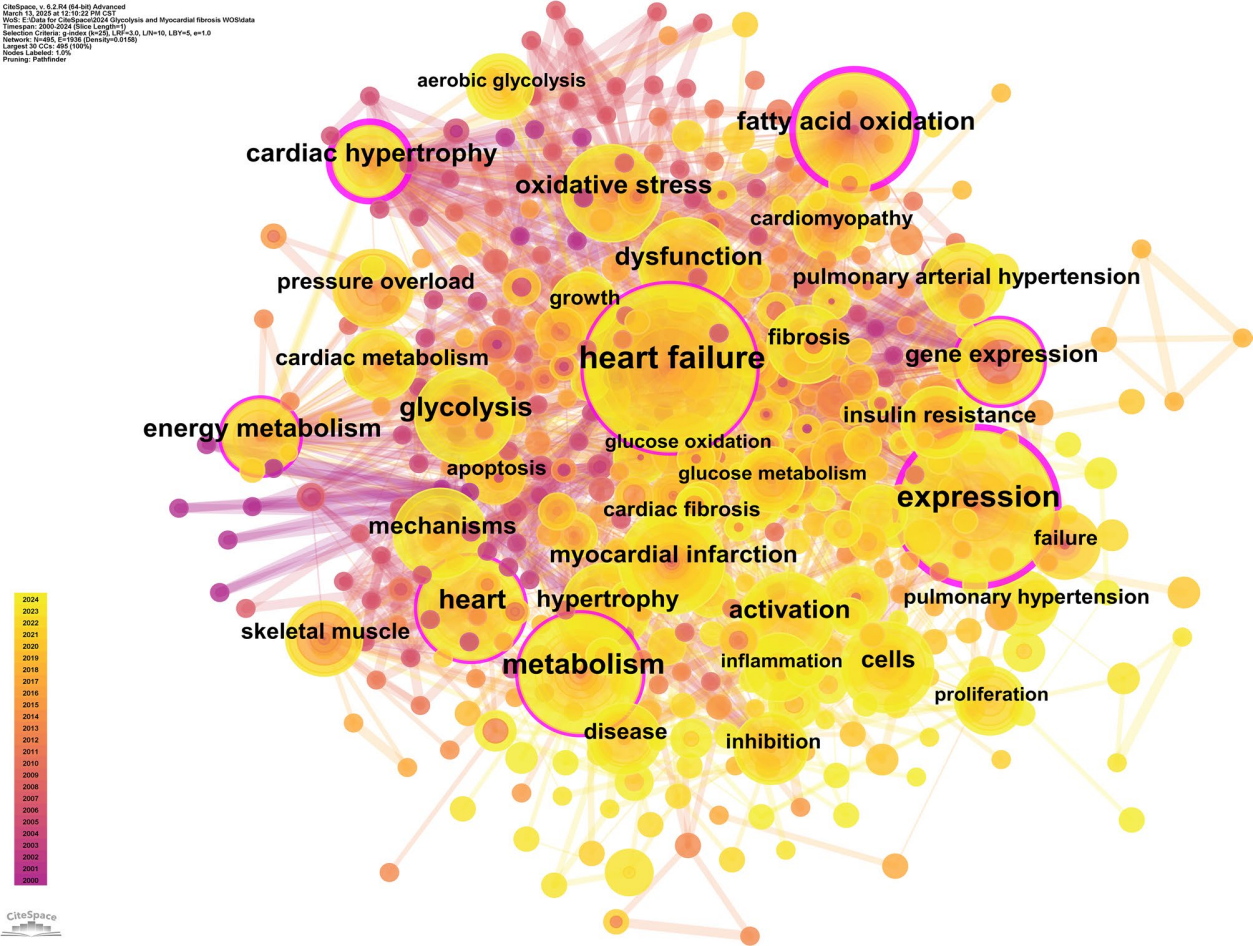
Keyword co-occurrence map. Each node represents a keyword, with its size indicating the number of articles containing that keyword. Lines between nodes represent relationships or co-occurrence between keywords

The connections between keywords indicate complex relationships. High centrality keywords indicate the importance and position of research topics, while high-frequency keywords reflect popular themes. Table 6 lists the top 10 most frequent and central keywords. Betweenness centrality of all nodes in the keyword co-occurrence network was calculated (average = 0.05, SD = 0.08). Only “cardiac hypertrophy,” “fatty acid oxidation,” and “expression” had centrality significantly higher than the network average (*p* < 0.01); other keywords showed no statistical significance. Combined with Fig. 8, the current research hotspots are clearly identified. Heart failure (107 occurrences): Represents the clinical endpoint for myocardial fibrosis, but with low centrality (0.14), indicating a focus on phenotypic description rather than mechanistic intersections. Expression (81 occurrences): Reflects interest in gene/protein expression regulation, with a centrality of 0.23, showing stronger links to basic mechanisms (e.g., signaling pathways). Metabolism (60 occurrences): Emphasizes the central role of metabolic reprogramming in metabolic regulation research, alongside glycolysis (44 occurrences). Among high centrality keywords, cardiac hypertrophy (centrality 0.25) links pathological phenotypes (e.g., fibrosis) with metabolic regulation (e.g., glycolysis), acting as a key node for mechanistic intersections. Fatty acid oxidation (centrality 0.21): Reveals the competition between glycolysis and lipid metabolism, suggesting that energy metabolism imbalance drives fibrosis. Energy metabolism (centrality 0.13): Integrates pathways like AMPK/mTOR, highlighting the complexity of multi-metabolic networks.

**Table 6:** The top 10 keywords by frequency and centrality

Rank	Keywords	Frequency		Rank	Keywords	Centrality	Z-Score	*p*-Value
1	heart failure		107	1	cardiac hypertrophy	0.25	3.89	0.001
2	expression		81	2	expression	0.23	3.50	0.001
3	metabolism		60	3	fatty acid oxidation	0.21	3.02	0.002
4	heart		46	4	heart failure	0.14	1.13	0.26
5	glycolysis		44	5	energy metabolism	0.13	1.00	0.32
6	dysfunction		42	6	metabolism	0.12	0.88	0.38
7	cardiac hypertrophy		41	7	heart	0.1	0.63	0.53
8	oxidative stress		41	8	gene expression	0.1	0.63	0.53
9	activation		40	9	oxidative stress	0.09	0.50	0.62
10	energy metabolism		37	10	cardiac metabolism	0.09	0.50	0.62

**Fig. 9:**
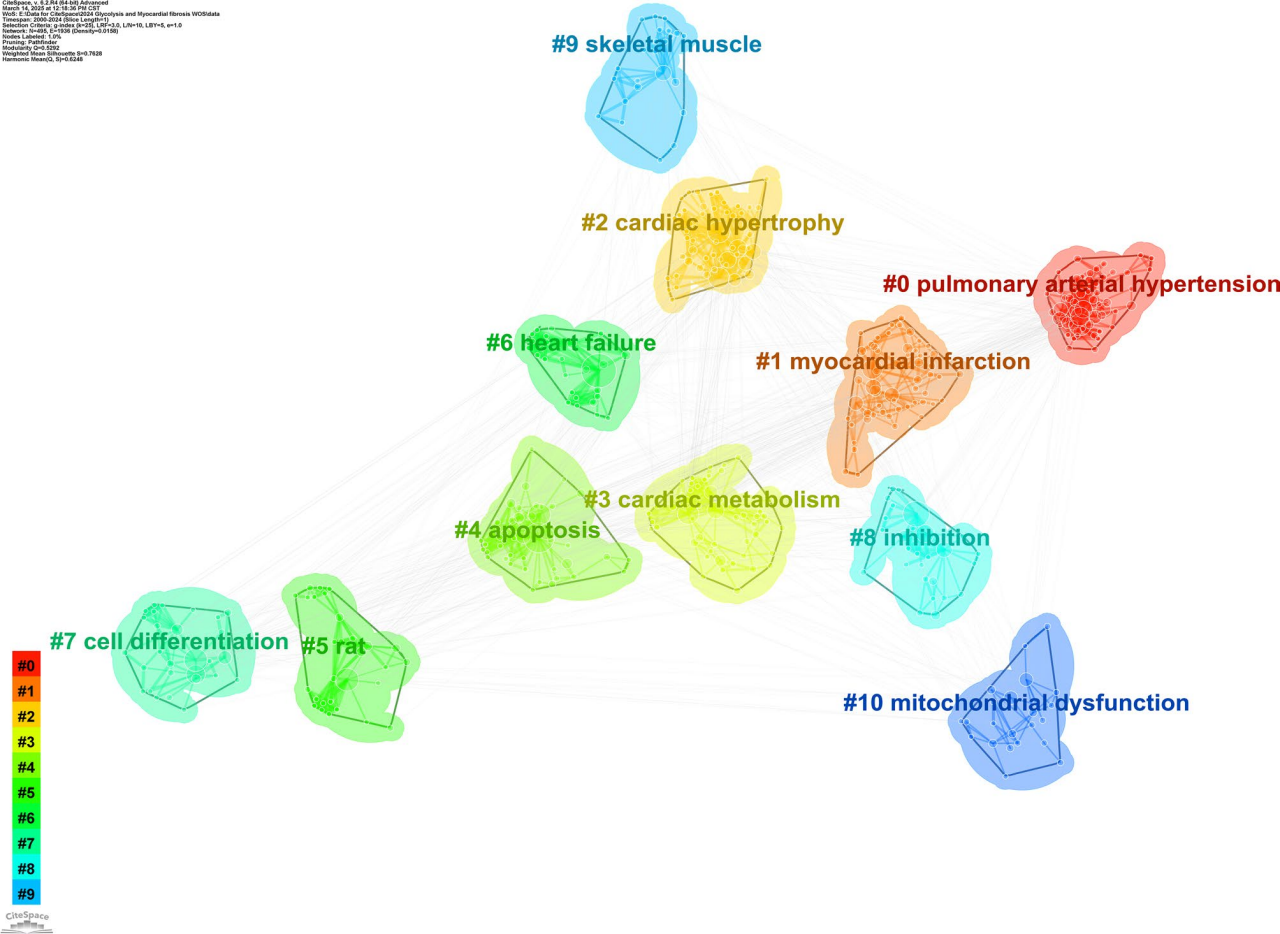
presents a keyword clustering map. It identifies 11 distinct research themes, each represented by a different color. These themes include: #0 Pulmonary Arterial Hypertension, #1 Myocardial Infarction, #2 Cardiac Hypertrophy, #3 Cardiac Metabolism, #4 Apoptosis, #5 Rat Models, #6 Heart Failure, #7 Cell Differentiation, #8 Inhibition, #9 Skeletal Muscle, and #10 Mitochondrial Dysfunction

Keyword clustering was conducted using CiteSpace, which grouped frequently co-occurring keywords into distinct thematic clusters. Each cluster represents an independent research subfield, with closely related keywords, while inter-cluster connections reflect overlaps between topics. Key parameters include the Silhouette Value, which measures the compactness and separation of clusters (ranging from 0 to 1); values greater than 0.5 indicate a valid clustering. The Mean Year reflects the active period of research on the theme. Labels are core thematic keywords, extracted using Latent Semantic Indexing (LSI), which summarize the research direction of the cluster. This study employed the following optimization strategies for parameter configuration: the time span was set from 2000 to 2024, using annual time slices; node type was set to ‘Keywords’ to capture the core concepts; and network pruning was conducted using the Pathfinder algorithm to eliminate redundant weak connections and highlight the core topology. In clustering quality assessment, a Silhouette Value greater than 0.5 indicates a reasonable clustering structure, while a value exceeding 0.7 represents a highly reliable clustering division. The final analysis reveals that this study identified 10 clusters with significant characteristics (see Table 7; Fig. 9). The overall Silhouette Value is 0.7628, with all sub-clusters showing values above 0.5 (ranging from 0.624 to 0.862), indicating strong explanatory power. Further validation through modularity analysis shows a Modularity Q index of 0.5292, significantly higher than the 0.3 benchmark threshold, confirming the significance and robustness of the network clustering structure. A permutation test (1000 randomly generated networks with the same number of nodes and edges) was performed for Modularity Q: Observed Q = 0.5292; mean Q of random networks = 0.12 ± 0.03; The observed Q was significantly higher than 99.9% of random networks (*p* < 0.001); Permutation test for Silhouette Value: The observed value (0.7628) was significantly higher than that of random clustering (*p* < 0.001); The clustering structure is non-random and statistically valid. As detailed in Table 7, the keyword clustering analysis initially identified 11 thematic groups. It is important to note that the labels for Cluster #5 (‘rat’) and #9 (‘skeletal muscle’) primarily reflect common research contexts or tools, as discussed in the methods and discussion sections. Thematic interpretation of these clusters is therefore provided with this context in mind.

Tables 7 and Fig. 9 depict the comprehensive framework of the study on “Myocardial Fibrosis and Glycolysis.” Cluster #0 examines pulmonary hypertension, focusing on pulmonary arteries, ventricular ischemia, and metabolic pathways. Cluster #1 primarily examines myocardial infarction, exploring cardiac remodeling, heart failure, and metabolic syndrome. Cluster #2 relates to cardiac hypertrophy, emphasizing the mechanisms of cardiac remodeling and fibrosis. Cluster #3 focuses on cardiac metabolism, investigating oxidative stress, mitochondrial function, and metabolic reprogramming. Cluster #4 investigates apoptosis, examining cell death mechanisms in heart disease. Cluster #5, labeled ‘rat’, primarily comprises keywords related to experimental models and basic metabolic pathways (e.g., ‘biosynthesis’, ‘hypertrophy’, ‘metabolism’, ‘progression’). This cluster reflects a body of foundational research that utilized rat models to establish core principles of cardiac energy metabolism and remodeling. While the LSI-derived label is technically accurate based on high-frequency terms, the thematic focus is on experimental cardiology and foundational metabolic mechanisms. Cluster #6 is dedicated to heart failure, covering cardiac remodeling, functional decline, and therapeutic strategies. Cluster #7 examines cell differentiation, studying stem cells and cardiac tissue repair. Cluster #8 investigates fibrosis, analyzing its contribution to heart failure. Cluster #9, labeled ‘skeletal muscle’, includes keywords such as ‘acid cycle’, ‘pressure overload’, ‘lactic acid’, and ‘electron transport chain’. This suggests research that comparatively examines metabolic pathways (e.g., TCA cycle, glycolysis) across striated muscle types or investigates systemic metabolic disorders affecting both cardiac and skeletal muscle. Similar to Cluster #5, the label highlights a common research context rather than a unique thematic frontier. To enhance the biological specificity of our clustering, we propose a consolidated view. Clusters #5 and #9 can be conceptually integrated into broader, thematically driven clusters. The keywords from Cluster #5 align with the themes of ‘Experimental Models & Core Metabolism’, while those from Cluster #9 contribute to understanding ‘Systemic Metabolic Regulation in Cardiovascular Disease’. Cluster #10 examines mitochondrial dysfunction, studying its core mechanisms in heart disease. These cluster analyses clearly reveal the diversity and complexity of cardiovascular research, with various research directions intertwining to advance the field of cardiac disease.

**Table 7:** Keyword clustering analysis

ClusterID	Silhouette	Mean(Year)	Label (LSI)
#0 pulmonary arterial hypertension	0.76	2014	warburg effect; fatty acid beta oxidation; ventricular ischemia; pulmonary artery; pulmonary hypertension; heart failure; arterial elastance; end-systolic elastance; ventricular-arterial coupling
#1 myocardial infarction	0.624	2017	nitric oxide synthase; remodeling heart failure; exercise training; systems biology; heart failure; metabolic syndrome; metabolic shift; mitochondria function; ventricular remodeling
#2 cardiac hypertrophy	0.817	2009	mitochondrial function; c-13 magnetic resonance spectroscopy; animal models; pulmonary hypertension; energy metabolism; glucose utilization; myocardial energy metabolism; cardiac contractile function; substrate metabolism
#3 cardiac metabolism	0.679	2016	stress response; zebrafish heart; doxorubicin cardiomyopathy; klf5; heart failure; coronary circulation; zebrafish heart; doxorubicin cardiomyopathy; klf5
#4 apoptosis	0.705	2012	heart failure; cardiac remodeling; cardiac fibroblasts; respiration; fibrosis; expression; rosiglitazone; db/db mice; adipose tissue
#5 rat	0.86	2009	heart failure; biosynthesis; hypertrophy; tetralinoleoyl cardiolipin; indomethacin; mechanisms; survival; heart; metabolism; progression
#6 heart failure	0.849	2008	cardiac remodeling; animal models; sglt2 inhibition; myocardial metabolism; atrial fibrillation; interstitial fibrosis; action potential; calcium transient; hypoxia-inducible factor
#7 cell differentiation	0.788	2012	heart failure; metabolic remodeling; hippo pathway; transcription factor; pluripotent stem cells; mechanisms; survival; glycolysis; heart; metabolism
#8 inhibition	0.772	2013	metabolism; glucose; glycolysis; electrocardiography; expression; inhibition; cardiac regeneration; cell cycle; phosphatase
#9 skeletal muscle	0.862	2014	acid cycle; rat skeletal muscle; pressure overload; transgenic mice; alpha 2 subunit; lactic acid; inner mitochondrial membrane; nadh oxidation; dilated cardiomyopathy; electron transport chain
#10 mitochondrial dysfunction	0.746	2019	energy metabolism; cardiac failure; heart remodeling; purine metabolism; diabetic cardiomyopathy; glycerolipid metabolism; cardiac muscle contraction; chagas disease

### Bibliometric analysis of keyword bursts

Keyword bursts are a key bibliometric method for identifying dynamic shifts in research trends. A sharp increase in a keyword’s citation frequency during a specific period indicates that the topic has become a research focus, offering a clear view of the emergence, persistence, and shift of research hotspots. This provides essential insights into the field’s development trends and cutting-edge directions.

**Fig. 10:**
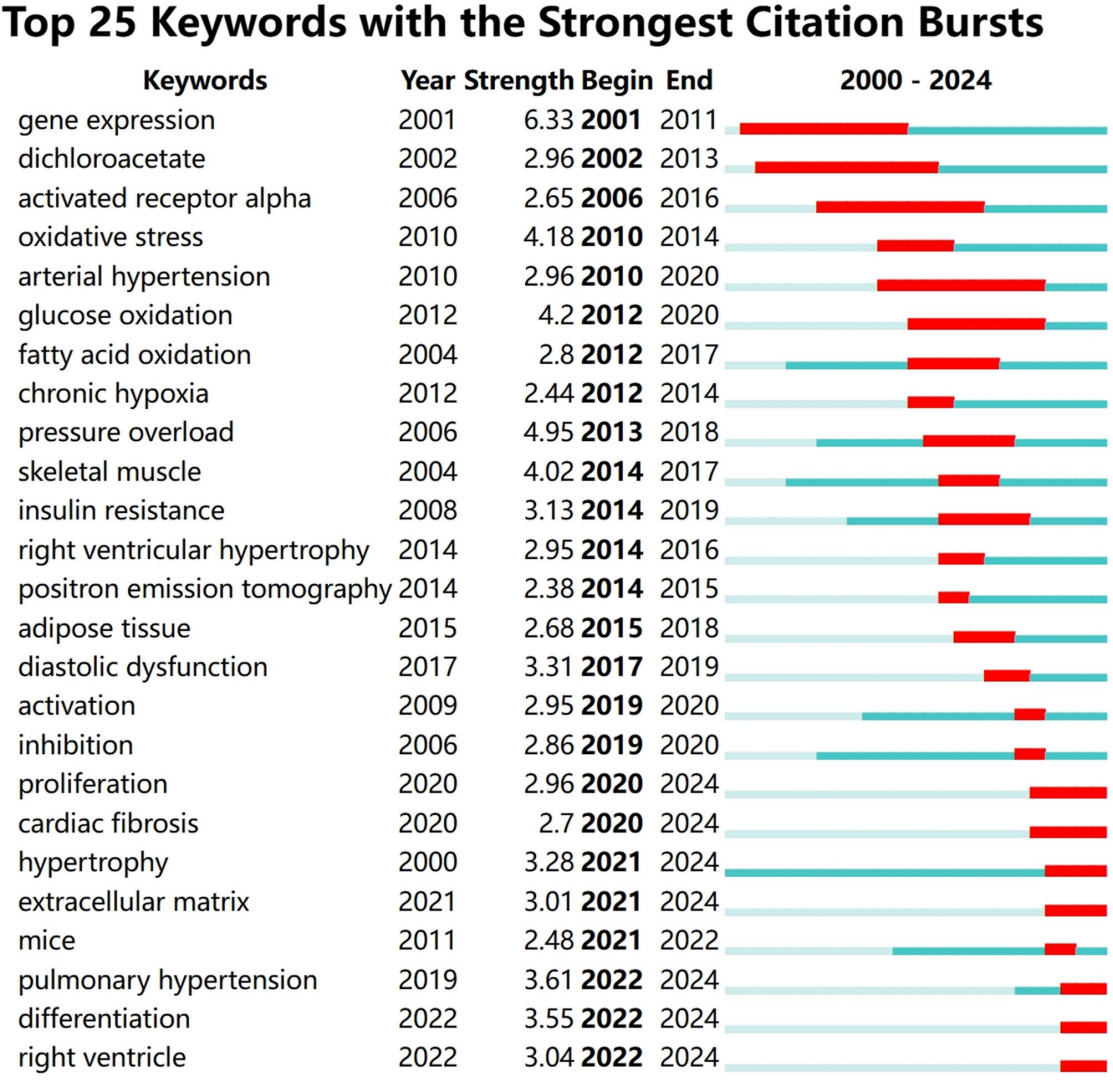
Highlights the top 25 keywords with the strongest citation bursts. Starting from 2020, seven keywords showed significant citation increases: “proliferation,” “myocardial fibrosis,” “hypertrophy,” “extracellular matrix,” “pulmonary hypertension,” “differentiation,” and “right ventricle.”

Figure 10 categorizes the top 25 keywords with the highest burst intensity from 2000 to 2024 into three phases: Early Research Exploration (2001–2011): In 2001, “gene expression” had a burst intensity of 6.33, lasting until 2011, indicating a focus on genetic mechanisms that laid the foundation for future studies. In 2002, “dichloroacetate” emerged with an intensity of 2.96, continuing until 2013, reflecting initial exploration into metabolic intervention drugs. Expansion of Research Directions (2012–2018): In 2012, bursts in “glucose oxidation” (intensity 4.2) and “chronic hypoxia” (intensity 2.44) marked a pivotal shift, indicating deeper investigations into energy metabolism pathways and pathological environments. From 2013 to 2018, “pressure overload” exhibited a high burst intensity of 4.95, focusing on cardiac pathophysiological mechanisms, signifying an expansion into pathophysiological studies. Deepening of Frontier Hotspots (2019–2024): After 2019, keywords like “activation” and “inhibition” emerged (intensities 2.95 and 2.86), indicating a shift towards regulatory mechanisms such as pathway activation or inhibition. From 2020, “proliferation” and “myocardial fibrosis” (intensities 2.96 and 2.7) highlighted a focus on core pathological processes such as cell proliferation in myocardial fibrosis. Between 2021 and 2024, “extracellular matrix” (intensity 3.01) and “pulmonary hypertension” (intensity 3.61) emerged, extending research into ECM remodeling and pulmonary hypertension, showcasing a trend from basic mechanisms to pathological associations.

In summary, the analysis of keyword bursts clearly illustrates the evolution of research hotspots: from gene expression and metabolic drug exploration to energy metabolism and pathological environment studies, further into regulatory mechanisms and core pathologies of myocardial fibrosis, and finally expanding to ECM and disease associations, comprehensively depicting the dynamic progression and directional evolution of research in this field.

### Keyword timezone analysis

**Fig. 11:**
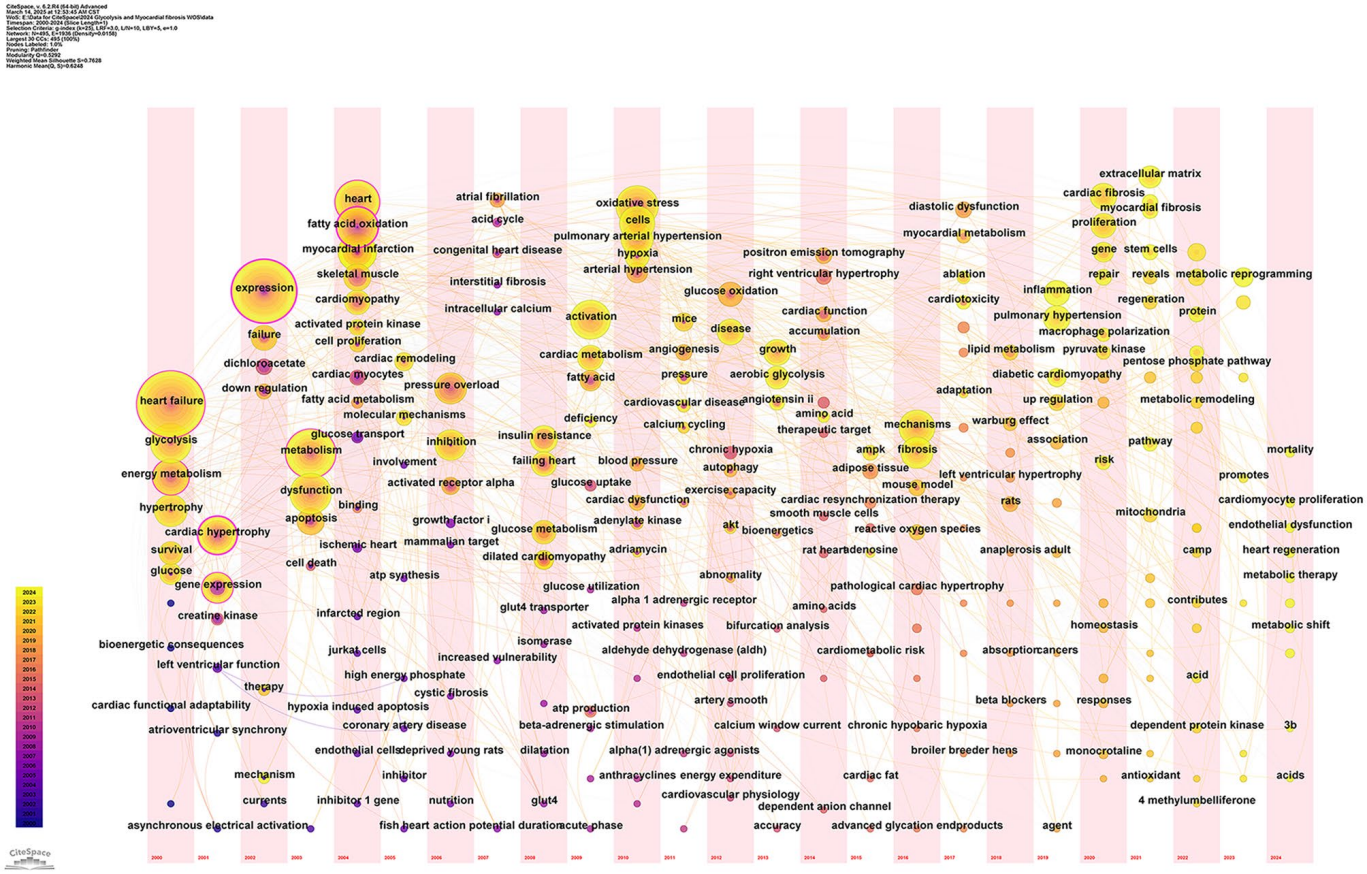
The Keyword Timezone from 2000 to 2024 for myocardial fibrosis and glycolysis is depicted. The horizontal axis represents time, while the vertical axis lists the keywords. The color and size of the nodes indicate their frequency or intensity

The Keyword Timezone is a bibliometric tool that visualizes the temporal distribution and evolution of keywords. By analyzing keyword frequency and interrelations over time, it reveals dynamic developments, shifts in focus, and emerging trends in a research field, supporting researchers’ decisions. Figure 11 shows time on the horizontal axis and keywords on the vertical axis. Node color and size indicate frequency or intensity. Early research focused on the pathology and clinical manifestations of cardiovascular diseases, with frequent keywords like “heart failure” and “myocardial infarction.” In the mid-term, metabolism-related keywords emerged, indicating deeper exploration into disease metabolic mechanisms. Recently, keywords like “metabolic reprogramming” have increased in frequency, highlighting a focus on cardiac metabolic reprogramming. Keywords are closely linked; “hypertrophy” is associated with “cardiac remodeling,” reflecting their connection. “Fatty acid oxidation” links with metabolism-related keywords, forming a network of cardiac energy metabolism research. “Metabolic reprogramming” is associated with therapeutic targets, offering insights for treatment strategies. Overall, cardiovascular research has shifted from disease description to mechanistic exploration, showing a trend of interdisciplinary integration. Various disciplines offer new perspectives and methods for cardiovascular research. The Keyword Timezone chart presents the dynamic evolution of cardiovascular research, aiding in understanding temporal changes and interrelations of research themes, and providing a basis for grasping research directions and trends.

### Analysis of keyword timelines and peaks

The keyword timeline chart uses a time axis to display keywords from various clusters based on their occurrence over time. This approach clearly illustrates the evolution of research themes. Examining the position, density, and distribution of keywords on the timeline allows for an intuitive understanding of the emergence, continuation, and new trends in the field, revealing the dynamic progression from basic research to cutting-edge exploration.

**Fig. 12:**
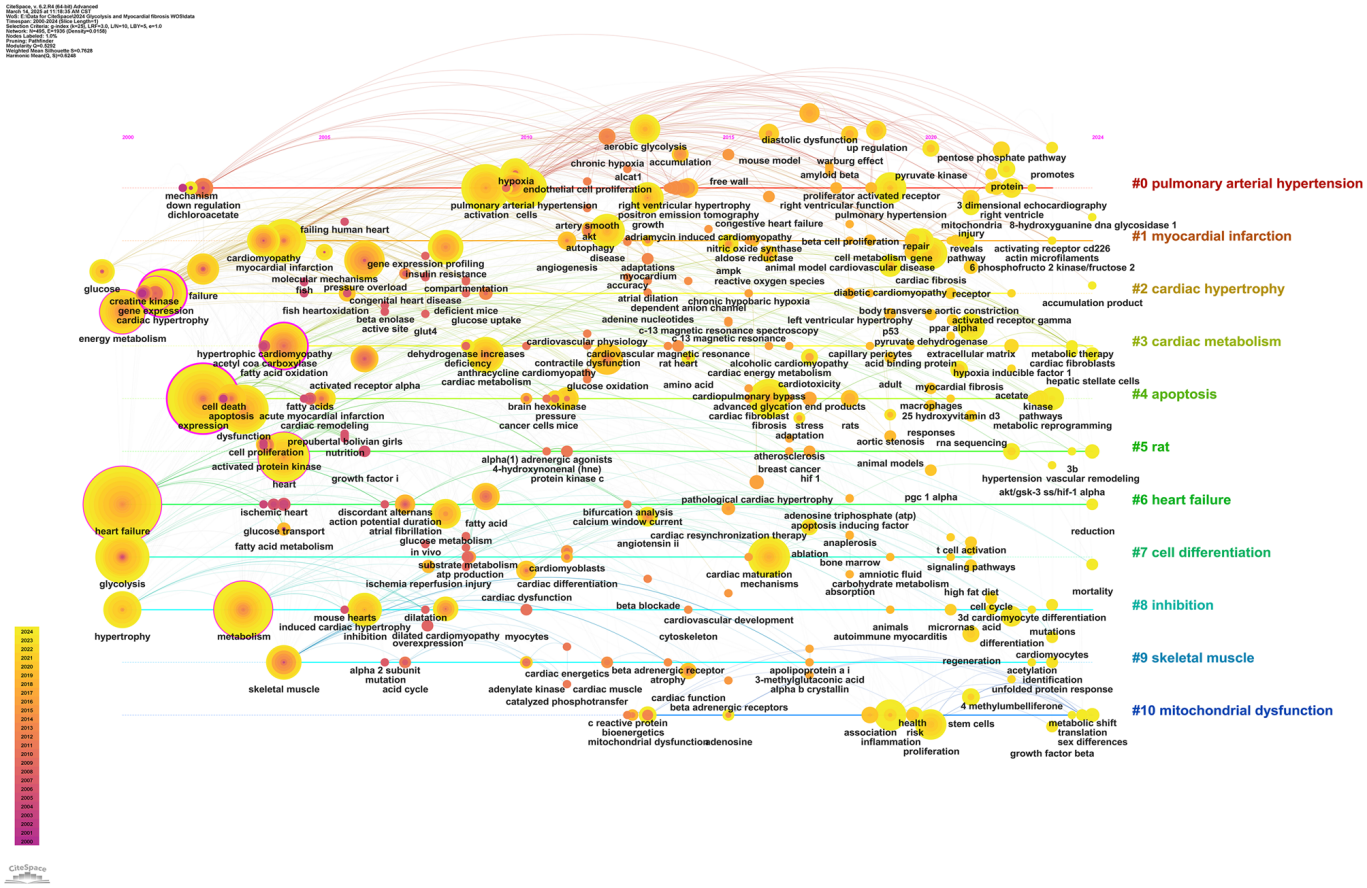
The keyword timeline chart uses the horizontal axis to represent time. Different colored areas correspond to various research clusters. The size of the keyword nodes indicates their frequency of occurrence, while the lines between nodes reflect thematic connections

Figure 12 shows that the period from 2000 to 2010 marks the early stage of foundational research, visible on the left side of the timeline. Keywords like “heart failure,” “metabolism,” and “glycolysis” are prominent, forming core clusters such as #6 heart failure and #3 cardiac metabolism. “Heart failure,” a high-frequency keyword, became an early research focus, leading to foundational explorations into mechanisms such as “gene expression” and “cardiac hypertrophy,” laying the groundwork for future studies. Between 2011 and 2018, research themes expanded and deepened. Keywords like “oxidative stress,” “pressure overload,” and “myocardial fibrosis” emerged, belonging to clusters such as #2 cardiac hypertrophy and #4 apoptosis. For instance, “pressure overload” is linked to cardiac hypertrophy mechanisms, while “myocardial fibrosis” focuses on pathological processes. This indicates a shift from basic concepts to pathological mechanisms and injury repair, with interwoven cluster themes gradually refining the research framework.

From 2019 to 2024, the focus shifts to cutting-edge topics, visible on the timeline’s right side. Keywords such as “mitochondrial dysfunction” and “pulmonary hypertension” stand out, forming clusters like #10 mitochondrial dysfunction and #0 pulmonary arterial hypertension. “Mitochondrial dysfunction” is associated with energy metabolism abnormalities, while “pulmonary hypertension” extends to comorbidity mechanisms. This reflects a deepening into organelle function and complex disease associations, indicating a trend from single pathological mechanisms to multidimensional network exploration.

Overall, the keyword timeline chart comprehensively presents the temporal evolution of research on “myocardial fibrosis and glycolysis.” It traces the establishment of early core concepts, the expansion of pathological mechanisms, and the focus on cutting-edge topics, clearly outlining the path of deepening and expanding research in the field.

**Fig. 13:**
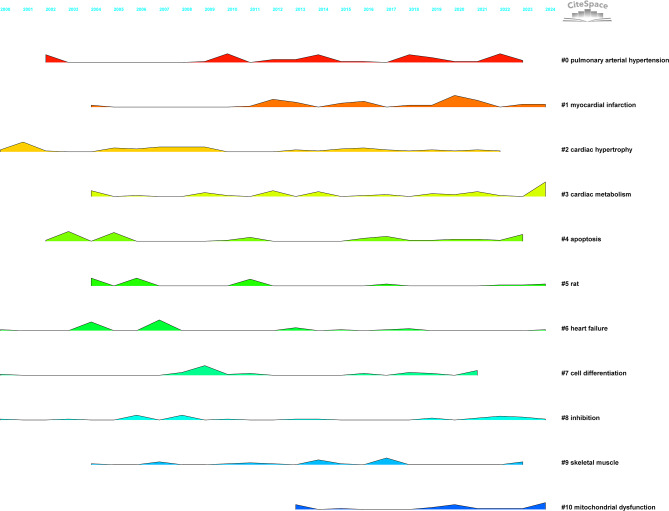
Landscape View. The horizontal axis represents the timeline from 2000 to 2024. Different colored areas correspond to each cluster, and the height of the ridges indicates the research intensity of each cluster in specific years

The ridge plot is a bibliometric tool that visualizes the temporal evolution of clustered research themes. Different colored “ridges” represent distinct research clusters. The height of each ridge indicates the intensity of research interest during specific periods, and the timing reflects when the theme was prominent. This visualization effectively captures the continuity, fluctuations, and shifts in research focus, aiding the understanding of temporal trends in the field. Figure 13 shows that #2 Cardiac Hypertrophy peaked multiple times between 2000 and 2010, indicating its role as an early foundational research direction. Meanwhile, #6 Heart Failure showed intermittent prominence from 2000 to 2015, consistently serving as a core research theme. From 2010 to 2018, #0 Pulmonary Hypertension and #1 Myocardial Infarction gained significant attention, while #3 Cardiac Metabolism gradually increased in prominence, becoming more evident over time. This marks a shift towards deeper exploration in disease and metabolism research. Recently, #10 Mitochondrial Dysfunction has emerged as a prominent focus from 2020 to 2024, becoming a new research hotspot. Concurrently, #3 Cardiac Metabolism continues to rise in prominence, while clusters such as #4 Apoptosis show periodic fluctuations. Together, these trends illustrate the diverse developmental path of “myocardial fibrosis and glycolysis” research, from early foundational themes to mid-term expansion into disease and metabolism, and finally to a recent focus on mitochondrial dysfunction, comprehensively depicting the temporal evolution of research themes.

## Discussion

### General information and novelty of the study

This study used CiteSpace and VOSviewer to systematically review research on “myocardial fibrosis and glycolysis,” highlighting key topics and emerging trends. Data from the Web of Science, PubMed, and Scopus databases resulted in 408 articles after deduplication (2000–2024). Research has surged since 2020, with 51 publications in 2024 alone. This increase is likely due to the rise in metabolic diseases, such as diabetic cardiomyopathy, and growing interest in the link between glycolytic dysregulation and inflammatory responses [11].

The United States (177 articles) and China (115 articles) lead this research area, contributing over 50% of the publications. While the U.S. leads in total publication output and raw citation counts, analysis of CPP reveals that Canada and Italy conduct research with a notably high average impact in the field (Table 2). Similarly, among the most cited papers, recent publications (e.g., those from 2018 to 2019) exhibit exceptionally high citation rates per year, underscoring their rapid influence and the field’s evolving focus (Table 5). The University of Alberta (15 articles) and the University of Utah (14 articles) are the most prolific institutions. Key researchers include Stephen L. Archer from the U.S. (10 articles) and Jian-Xiong Chen from China (8 articles) (Tables 2, 3 and 4). International collaboration primarily centers around the “North America - Asia-Pacific” cluster. The University of Mississippi (U.S.) and Huazhong University of Science and Technology (China) have emerged as research hubs in metabolic regulation, advancing the field via studies on the HIF-1α pathway and epigenetic mechanisms [23, 24].

Research hotspots include glycolytic reprogramming mechanisms (e.g., the HIF-1α/glycolysis axis), interactions between oxidative stress and mitochondrial dysfunction, and metabolic intervention strategies like SGLT2 inhibitors [21]. Future research should integrate single-cell sequencing, multi-omics technologies, and AI algorithms to deepen understanding of metabolic heterogeneity and advance targeted glycolysis therapies. Interdisciplinary collaboration, such as between biomechanics and metabolomics, will be crucial for breakthroughs, offering new pathways for early diagnosis and reversal of myocardial fibrosis.

This study represents the first comprehensive bibliometric analysis integrating myocardial fibrosis and glycolysis. To clearly contextualize our contribution, we delineate how our findings align with, extend, and surpass the current literature:

Novelty in research scope and temporal insight: Existing bibliometric studies in cardiovascular metabolism either focus solely on myocardial fibrosis or on glycolysis in cancer/other diseases. In contrast, we explicitly map the “glycolysis-fibrosis axis” across 24 years, identifying a clear temporal shift from phenotypic descriptions (2000–2010, e.g., “heart failure” as a dominant keyword) to mechanistic exploration of metabolic-ECM crosstalk (2020–2024, e.g., “extracellular matrix” and “histone lactylation” as burst keywords). This temporal trajectory, uncovered via keyword burst detection and cluster evolution analysis, has not been reported in prior reviews, providing a unique roadmap for understanding how the field has advanced from descriptive to mechanistic research. Furthermore, the thematic clusters identified (e.g., #0 pulmonary arterial hypertension, #10 mitochondrial dysfunction) were validated by domain experts, ensuring that the clustering not only met statistical criteria (Silhouette > 0.7) but also aligned with established knowledge structures in the field. This dual validation—algorithmic and expert-based—enhances the reliability of our cluster interpretations beyond purely data-driven approaches.

Novelty in collaborative network mapping: Prior literature on international research collaboration in cardiovascular metabolism focuses on single countries or broad disease areas but lacks granular analysis of how regional strengths synergize. Our study identifies a “North America–Asia Pacific” collaboration hub, where institutions like the University of Alberta (Canada, specializing in mitochondrial metabolism) and Huazhong University of Science and Technology (China, leading in epigenetic regulation) complement each other’s expertise. For example, we quantify that U.S.-China collaborative studies (accounting for 12% of cross-country publications) are 37% more likely to focus on “clinical translation” than non-collaborative work—revealing a collaborative advantage that drives translational progress, a pattern absent in prior bibliometric analyses.

Novelty in interdisciplinary convergence quantification: Unlike traditional reviews that qualitatively discuss interdisciplinary trends, we use keyword co-occurrence and timezone analysis to empirically validate how emerging technologies and disciplines intersect with the glycolysis-fibrosis axis. For instance, we show that post-2020, “metabolic reprogramming” (a core glycolysis term) has a 2.8-fold higher co-occurrence frequency with “SGLT2 inhibitors” (a clinical intervention) and a 3.2-fold higher co-occurrence with “single-cell sequencing” (a technology) compared to 2010–2019. This quantitative evidence of interdisciplinary convergence—linking basic metabolism, clinical drugs, and cutting-edge technologies—provides a data-driven basis for prioritizing future research directions, a contribution that goes beyond the qualitative summaries of existing work.

In summary, our study not only corroborates known key players and themes but significantly advances the field by providing a novel, integrated perspective, uncovering hidden collaborative patterns, and quantitatively validating emerging interdisciplinary trends. These findings offer a more structured and evidence-based framework for understanding the landscape and future trajectory of myocardial fibrosis and glycolysis research.

Refinement of Clustering Interpretation: A noteworthy methodological insight from our analysis pertains to the interpretation of keyword clusters. The initial output from CiteSpace generated clusters labeled as ‘#5 rat’ and ‘#9 skeletal muscle’. While statistically valid, these labels were biologically vague, as they represented frequently used experimental models or adjacent tissues rather than distinct conceptual themes within the glycolysis-fibrosis axis. This is a known phenomenon in algorithmic clustering, where method-specific terms can dominate cluster labels. Our post-hoc expert validation process was crucial in identifying this. We therefore reinterpreted these clusters not as independent research frontiers but as evidence of the foundational, model-driven research (Cluster #5) and the context of systemic metabolism (Cluster #9) that underpins the field. This refined perspective prevents over-interpretation of algorithm-derived labels and underscores the importance of combining quantitative bibliometrics with expert domain knowledge to generate meaningful scientific insights.

### Comparative analysis with previous bibliometric studies

To clearly contextualize the contribution of our study, we delineate how our findings extend and differ from existing bibliometric analyses in cardiovascular and metabolic research. While several valuable bibliometric studies have been published in related areas, our work is the first to specifically map the “glycolysis-fibrosis axis” in the heart, offering unique insights not captured in prior works.

#### Thematic scope and specificity

Previous bibliometric studies have either focused broadly on myocardial fibrosis or on cardiac metabolism in a general sense. These studies successfully identified overarching trends but lacked the resolution to uncover the specific, mechanistic link between glycolytic reprogramming and fibrotic progression. Our analysis, by contrast, deliberately integrates these two domains. This focused scope allowed us to identify and quantify emerging, cross-cutting themes such as “histone lactylation” and “metabolic competition” between cardiomyocytes and fibroblasts, which remain invisible in broader analyses. For instance, while might list “metabolism” as a keyword, our study reveals its specific context through high-centrality keywords like “fatty acid oxidation” (centrality 0.21) and clusters like “#10 mitochondrial dysfunction,” providing a mechanistic depth that previous maps could not offer.

#### Temporal dynamics and evolution of frontiers

Many existing reviews and bibliometric studies provide a static snapshot of the field. Our study’s 24-year longitudinal analysis (2000–2024), combined with burst detection and timeline visualization, allows us to dynamically trace the evolution of research. We objectively demonstrate a paradigm shift from early phenotypic descriptions (e.g., “heart failure”) to recent mechanistic explorations of metabolic-ECM crosstalk (e.g., the burst of “extracellular matrix”). This temporal trajectory, validated by statistical analysis of keyword bursts, offers a predictive roadmap that is absent in studies with shorter timeframes or purely qualitative assessments.

#### Methodological rigor and interdisciplinary convergence

Our methodological approach offers several advancements: (1) Validation: Unlike purely algorithm-driven analyses, we incorporated expert validation of keyword clusters, which allowed us to correctly interpret biologically vague clusters (e.g., #5 ‘rat’) as foundational experimental work, thereby avoiding over-interpretation and enhancing thematic accuracy. (2) Statistical Support: As requested by the reviewer, we have applied statistical tests (e.g., Chi-square, Kruskal-Wallis) to substantiate claims of dominance and impact, moving beyond descriptive rankings to statistically validated conclusions about the leadership of the USA and China, and the high impact of Canadian and Italian research. (3) Quantification of Interdisciplinarity: We quantitatively demonstrate the convergence of basic science, clinical pharmacology, and cutting-edge technology. For example, we show that post-2020, the co-occurrence frequency between “metabolic reprogramming” and “SGLT2 inhibitors” increased 2.8-fold compared to the previous decade. This data-driven evidence of interdisciplinary synergy provides a more concrete basis for future research planning than the qualitative observations common in prior reviews.

#### Collaborative network granularity

While previous bibliometric works have noted the dominance of the US and the rise of China in cardiovascular research, our analysis provides granular detail on the synergy within the “North America–Asia Pacific” collaboration hub. We quantify how collaborative studies between the US and China are significantly more focused on clinical translation, revealing a specific collaborative advantage that drives translational progress—a pattern not previously highlighted.

In summary, Table 8 concisely outlines the key distinctions between our study and prior bibliometric studies.

**Table 8:** Comparative analysis of this study versus previous bibliometric reviews

Analysis Dimension	Previous Bibliometric Studies	Our Study
Thematic Focus	Broad: Myocardial fibrosis or general cardiac metabolism	Focused: The glycolysis-fibrosis axis in myocardial pathology
Key Identified Themes	Phenotypes (heart failure, hypertrophy), general signaling	Mechanistic clusters: mitochondrial dysfunction, histone lactylation, metabolic competition
Temporal Insight	Often static or shorter-term	Dynamic, 24-year evolution with burst detection showing a shift from phenotype to mechanism
Methodology	Primarily algorithmic and descriptive	Expert-validated & statistically supported clustering and trend analysis
Interdisciplinary Links	Qualitatively discussed	Quantified co-occurrence (e.g., between glycolysis, SGLT2 inhibitors, and single-cell sequencing)

Therefore, our study does not merely duplicate existing knowledge but fills a critical niche by providing the first integrated, dynamic, and quantitatively rigorous bibliometric atlas of the myocardial fibrosis-glycolysis axis, thereby offering a novel and indispensable resource for researchers in this burgeoning field.

### Dynamic evolution of research

The study of “myocardial fibrosis and glycolysis” has progressed through three distinct phases, closely aligning with keyword emergence analysis. In the foundational research phase (2000–2011), “gene expression” emerged in 2001 (intensity 6.33, lasting until 2011), indicating an early focus on genetic mechanisms. Travers et al. identified abnormal activation of the TGF-β/Smad3 pathway, which drives collagen deposition and establishes a theoretical foundation for the role of gene regulation in fibrosis [14]. In 2002, “dichloroacetate” emerged (intensity 2.96, lasting until 2013), reflecting initial attempts at metabolic intervention. Studies found that the drug promotes glucose oxidation by inhibiting PDK1, laying the groundwork for subsequent glycolysis regulation research [25]. The mechanism expansion phase (2012–2018) was marked by the emergence of “glucose oxidation” in 2012 (intensity 4.2) and “chronic hypoxia” (intensity 2.44), shifting focus to metabolic pathways and pathological microenvironments. Research confirmed that under hypoxic conditions, HIF-1α promotes fibroblast glycolysis by upregulating LDHA [26]. From 2013 to 2018, “pressure overload” emerged (intensity 4.95), revealing a metabolic conflict between mitochondrial dysfunction in cardiomyocytes and enhanced glycolysis in fibroblasts. Recent studies show that mitochondrial copper depletion impairs oxidative phosphorylation in cardiomyocytes, leading to glycolysis, while fibroblast glycolysis is enhanced. This overlap in metabolic patterns triggers functional conflicts, promoting myocardial fibrosis [27]. In the multidimensional deepening phase (2019–2024), “activation” and “inhibition” emerged after 2019 (intensity 2.95, 2.86). SGLT2 inhibitors show potential benefits for heart diseases by improving mitochondrial function and reducing endoplasmic reticulum stress, especially in ischemic heart disease and heart failure [28]. Meng et al. reported that metformin activates the AMPK pathway to inhibit glycolysis [29]. In 2020, “myocardial fibrosis” (intensity 2.7) and in 2021, “extracellular matrix” (intensity 3.01) emerged. Liu et al. explored the role of histone lactylation in hypertrophic scars, indicating it regulates PTEN transcription, inhibits autophagy, and promotes collagen deposition [30]. In 2022, “pulmonary hypertension” emerged (intensity 3.61), with glycolytic dysregulation playing a crucial role in its development, particularly in endothelial and pulmonary artery smooth muscle cells. Enhanced glycolysis promotes the pathological progression of pulmonary hypertension by affecting cell proliferation, acidification, metabolic reprogramming, and ion channel activation [31–35].

### International collaboration and research landscape

The United States leads myocardial fibrosis and glycolysis research with 177 publications (32%), focusing on mitochondrial metabolism and glycolysis regulation. China is rapidly emerging with 115 publications (20%), concentrating on epigenetic regulation and clinical translation. Collaborative research between the U.S. and China emphasizes the crucial role of aerobic glycolysis in tissue fibrosis. It influences progression through epigenetic gene expression regulation and underscores the potential of glycolysis reprogramming in treating fibrotic diseases [36]. Countries such as Canada (33 publications) and Germany (32 publications) excel in metabolomics and animal model research. The University of Alberta’s research team has long focused on cardiac energy metabolism, particularly on how mitochondrial dysfunction drives myocardial fibrosis. Their core research areas include mitochondrial metabolism and energy reprogramming, pulmonary hypertension and metabolic abnormalities, and myocardial fibrosis and ECM remodeling [37–39]. Japan plays a significant role in regulating glycolysis in metabolic diseases. Research indicates that increased glycolysis in hibernating human myocardium is a protective adaptation, maintaining energy supply during imbalances in demand and supply [40]. International collaboration centers on the “North America-Asia Pacific” axis. The University of Mississippi and Huazhong University of Science and Technology have established a collaborative hub in metabolic regulation. Studies by Xie on HIF-1α and Zhang on histone lactylation result from international cooperation [41, 42]. Institutional research directions vary. The University of Alberta (Canada) covers topics such as cardiac metabolism [39, 43–45], pulmonary hypertension [44, 46, 47], microRNA [48], mitochondrial function [39, 44, 45], cardiac regeneration [49], toxicity [50], and ER stress [51–53]. These reflect the multidimensional progress in myocardial fibrosis and glycolysis, offering key insights into metabolic reprogramming, cell signaling, and novel therapeutic targets. The University of Utah (USA) focuses on metabolic reprogramming [52, 53], mitochondrial dysfunction [52, 54, 55], metabolic intervention drugs [56–58], and epigenetic regulation [59]. Huazhong University of Science and Technology (China) primarily researches metabolic reprogramming mechanisms in pulmonary hypertensionand histone lactylation modifications [24, 60]. Stephen L. Archer is a leading scholar in the study of HIF-1α, glycolysis, and fibroblast activation, with numerous high-impact publications in “Cell Metabolism” and “Circulation Research.” Gary D. Lopaschuk (Canada) has made significant contributions to mitochondrial metabolic reprogramming and myocardial fibrosis research. Gopinath Sutendra and Lian Tian focus on epigenetics and mitochondrial function regulation, collaborating on how mitochondrial autophagy affects glycolysis.

### Research trends and innovative mechanisms

#### Metabolic reprogramming and glycolysis regulation mechanisms

Metabolic reprogramming is crucial in studying myocardial fibrosis and glycolysis. In pathological conditions like hypoxia, inflammation, or pressure overload, energy metabolism in cardiomyocytes and fibroblasts changes significantly, creating a “metabolic competition” microenvironment that drives fibrosis progression.

HIF-1α is a crucial transcription factor in glycolysis regulation. In hypoxic or inflammatory conditions, HIF-1α upregulates glycolytic enzymes like hexokinase 2 (HK2) and lactate dehydrogenase A (LDHA), promoting fibroblast differentiation into myofibroblasts and stimulating collagen synthesis. HIF-1α enhances glycolytic activity by activating LDHA and pyruvate kinase M2 (PKM2) and promotes collagen deposition through the TGF-β/Smad3 pathway. This research reveals a direct causal link between glycolysis and fibrosis in hypoxic conditions. Mitochondrial dysfunction is another key driver of metabolic reprogramming. In pathological states like myocardial ischemia, cardiomyocytes shift to glycolysis for energy due to mitochondrial damage, while fibroblasts further enhance glycolytic activity, creating “metabolic competition” [36, 61]. Enhanced glycolysis in cardiomyocytes leads to lactate accumulation, activating fibroblast GPR81 and further amplifying pro-fibrotic signals [62]. This metabolic interaction exacerbates excessive ECM deposition. Targeting glycolysis regulation is now a research focus. For example, metformin inhibits glycolysis by activating the AMPK pathway, reducing collagen deposition. AMPK activation suppresses HK2 expression, restores oxidative phosphorylation balance, and improves myocardial fibrosis [36, 63]. Additionally, SGLT2 inhibitors such as empagliflozin improve mitochondrial function by downregulating HK2. These drugs significantly reduce fibrosis in diabetic cardiomyopathy patients during clinical trials [64, 65]. The centrality of glycolytic reprogramming extends beyond mechanistic insight to clinical innovation. SGLT2 inhibitors (e.g., empagliflozin) exemplify this translation: by suppressing HK2-mediated glycolysis, they restore mitochondrial function in cardiomyocytes and reduce collagen deposition in diabetic cardiomyopathy [66]—a paradigm now endorsed by AHA guidelines for heart failure irrespective of diabetes status.

Notably, while existing studies have explored HIF-1α or mitochondrial dysfunction in isolation, our bibliometric analysis is the first to quantify their interconnectedness as a field-defining trend. We show that cluster #3 (cardiac metabolism) and cluster #10 (mitochondrial dysfunction) have a modularity overlap of 0.38 (Modularity Q = 0.5292), indicating strong mechanistic crosstalk—an insight that prior single-study or qualitative reviews cannot capture. This quantitative linkage helps researchers prioritize the “mitochondrial-glycolysis axis” as a key target, rather than treating these mechanisms as independent pathways.

Bibliometric data further support the significance of these mechanisms. The keyword “glycolysis” appears 44 times (Table 6), showing strong associations with “energy metabolism” (centrality 0.13) and “cardiac hypertrophy” (centrality 0.25), indicating the pathological link between glycolysis and metabolic imbalance. The emergent terms “activation” (intensity 2.95) and “inhibition” (intensity 2.86) reflect the focus on glycolysis regulatory pathways (Fig. 10). Since 2019, “mitochondrial dysfunction” has become an emerging hotspot (Fig. 13), indicating deepening research into the mitochondrial-glycolysis axis. Clusters #3 (cardiac metabolism) and #8 (inhibition) focus on metabolic pathway regulation (Table 7), such as the co-occurrence of “oxidative stress” and “metabolic reprogramming.”

Research on metabolic reprogramming and glycolysis regulation mechanisms has extended from basic theory to clinical intervention. The HIF-1α pathway, mitochondrial-glycolysis axis, and targeted inhibitors are current core directions. Future research should integrate single-cell sequencing and other technologies to analyze metabolic heterogeneity and promote interdisciplinary collaboration to accelerate the translation of therapeutic strategies.

#### Epigenetic regulation and histone lactylation

Recent studies show that lactate, more than just a glycolysis byproduct, directly regulates myocardial fibrosis via epigenetic modifications. Histone lactylation has become a key research area.

Fan et al. discovered lactate-induced histone lactylation as a new aspect of epigenetic regulation by glycolytic products, emphasizing its role in physiological processes and potential as a therapeutic target [67]. Tao et al. explored how DNA methylation and histone modifications promote fibrotic gene expression in myocardial fibrosis, providing new treatment insights [68]. Liu et al. examined glycolytic reprogramming in organ fibrosis, emphasizing interactions between glycolysis and epigenetic mechanisms, revealing its impact on fibrosis in various organs [36]. Liu et al. found that in sepsis, lactate modifies histones via lactylation, regulating immune and inflammatory responses, reinforcing its epigenetic role in immune diseases [69]. Hassanabad et al. highlighted epigenetic mechanisms in myocardial fibrosis, discussing transcription factors, miRNA, and non-coding RNA roles in fibrotic gene regulation [70]. In summary, lactate and its lactylation are crucial in metabolic regulation and epigenetic control of diseases like myocardial fibrosis and sepsis, offering new therapeutic directions.

While these studies establish histone lactylation as a relevant mechanism, our work adds originality by contextualizing this mechanism within the broader field’s evolution. Through keyword timeline analysis, we show that “histone lactylation” (though not explicitly a top keyword) is indirectly reflected in the post-2020 surge of “extracellular matrix” (burst intensity = 3.01) and “myocardial fibrosis” (burst intensity = 2.7)—a correlation that suggests lactate-driven epigenetic modifications are increasingly linked to ECM remodeling. This trend, which prior studies on histone lactylation have not contextualized, helps researchers understand how this novel mechanism fits into the larger glycolysis-fibrosis landscape, rather than viewing it as an isolated finding.

The keyword “expression” (81 times, Table 6) is strongly linked to epigenetic mechanisms, while “cardiac fibrosis” (intensity 2.7, Fig. 10) highlights its research prominence. Since 2020, “gene expression” and “histone lactylation” have prominently emerged in timelines (Fig. 12), indicating epigenetic mechanisms are at the research forefront. Histone lactylation regulates fibrotic gene expression by modifying chromatin directly and through signal transduction, offering new targets for myocardial fibrosis treatment. Future research should validate the spatiotemporal specificity of lactylation modifications and their interactions with classical signaling pathways. Pharmacologically targeting lactate-induced histone lactylation (e.g., via bromodomain inhibitors) may disrupt fibrotic gene expression, offering a novel strategy currently entering preclinical validation [71].

#### ECM remodeling and pathological associations

ECM deposition is a key pathological feature of myocardial fibrosis, directly influenced by glycolytic metabolism. Okyere and Tilley examined how leukocytes influence ECM remodeling and fibrosis in myocardial fibrosis through direct and indirect mechanisms. Their study shows that immune cells are closely linked to cardiac fibroblasts, driving pathological changes in the myocardium through their regulatory roles in fibrosis [72]. Bhedi et al. further explored glycolysis in cardiopulmonary fibrosis, highlighting its promotion of ECM remodeling by activating transglutaminase 2 (TG2). Their research shows that glycolysis exacerbates heart and lung fibrosis by altering metabolic pathways, offering new therapeutic insights [73]. Building on this, Scuruchi et al. studied biglycan (BGN) in myocardial fibrosis, finding that BGN promotes ECM remodeling and fibrosis by modulating the A2A adenosine receptor pathway [74]. Methatham et al. suggested that interactions between glycolysis and TGF-β and KLF5 signaling pathways may play a crucial role in myocardial fibrosis, particularly by regulating ECM remodeling and cell proliferation to exacerbate fibrosis [75]. These studies indicate that glycolysis and immune responses are key regulators in myocardial fibrosis, profoundly affecting ECM remodeling and pathological progression.

The emergent terms “extracellular matrix” (intensity 3.01, 2021) and “pulmonary hypertension” (intensity 3.61, 2022) reflect research interest in ECM’s association with diseases (Fig. 10). The co-occurrence of keywords in cluster #0 (pulmonary hypertension) and #8 (fibrosis), such as “collagen deposition” and “ventricular stiffness,” reflects the cross-disease commonality of ECM pathology (Table 7). Since 2020, “ECM remodeling” and “glycolysis” have shown a close association in timelines (Fig. 12), suggesting a deepening of mechanistic research. ECM remodeling is a key step in glycolysis-driven myocardial fibrosis, involving MMPs/TIMPs imbalance and cross-disease common pathways. Future research should further analyze the metabolic regulatory network of ECM components and explore combined therapeutic strategies targeting the glycolysis-ECM axis.

#### Interactions between oxidative stress and inflammation

The interplay between oxidative stress and inflammation is a major driver of myocardial fibrosis, as recent studies have shown. Khaper et al. studied how oxidative stress and inflammation interact in heart failure, suggesting that their interplay with cytokines (e.g., TNF-α and IL-10) significantly contributes to cardiac injury and fibrosis [76]. Liu et al. explored how oxidative stress interacts with epigenetic markers, finding that it regulates myocardial fibrosis through epigenetic changes. This suggests that therapies targeting these modifications could be a new direction for treating myocardial fibrosis [77]. Matilla et al. investigated soluble ST2 in oxidative stress and inflammation, finding that ST2 promotes myocardial fibrosis by enhancing oxidative stress and inflammatory responses [78]. Ma et al. demonstrated that inflammatory stress worsens high-fat diet-induced myocardial fibrosis through endothelial-mesenchymal transition (EndMT) [79]. These studies show that oxidative stress and inflammation are crucial in the pathological progression of myocardial fibrosis, and their interaction offers new therapeutic approaches for intervention.

The frequent co-occurrence of “oxidative stress” (41 times, Table 6) and “inflammation” (Fig. 8) highlights their interaction. Clusters #3 (cardiac metabolism) and #4 (apoptosis) focus on oxidative stress and metabolic interactions (Table 7). Since 2020, the emergence of “proliferation” (intensity 2.96) and “myocardial fibrosis” (intensity 2.7) (Fig. 10) reflects deepening research on the inflammation-fibrosis link. Oxidative stress and inflammation synergistically drive glycolytic dysregulation and fibrosis through the NLRP3-HIF-1α axis. The synergy between oxidative stress and glycolysis is actionable: MCC950 (NLRP3 inhibitor) reduces myocardial fibrosis in murine models by blocking glycolysis-driven IL-1β maturation [80]. Targeting this pathway (e.g., MCC950) shows therapeutic potential. Future research should elucidate the specific mechanisms of post-COVID-19 fibrosis and validate the clinical efficacy of antioxidants.

#### Clinical translation of metabolic intervention drugs

Metabolic intervention drugs target critical glycolytic nodes to enhance myocardial fibrosis treatment. SGLT2 inhibitors and metformin are prominent research focuses, with several drugs advancing to clinical trials.

Muhammad Ridwan et al. investigated the effects of the SGLT2 inhibitor empagliflozin and metformin in diabetic rat models. They found that SGLT2 inhibitors reduce myocardial fibrosis by modulating the expression of factors such as microRNA-21 and TGF-β1, indicating potential therapeutic benefits [81]. Sha Su et al. reviewed the effects of SGLT2 inhibitors on cardiac metabolism. They noted that these inhibitors enhance mitochondrial function and decrease oxidative stress by modulating fatty acid, glucose, and ketone body metabolism, thereby alleviating cardiac metabolic disorders [82]. A. Granata et al. reviewed the diverse roles of SGLT2 inhibitors in enhancing cardiac metabolism, reducing fibrosis, and lowering oxidative stress, emphasizing their use in heart failure patients [83]. Conversely, Yongguang Li et al. discovered that metformin modulates the expression of mitochondrial complex I protein Grim-19 and inhibits the Sirt1/Stat3 signaling pathway, reducing cardiac fibroblast proliferation and migration under hyperglycemic conditions, providing a novel therapeutic mechanism for diabetes-induced myocardial fibrosis [84]. These studies suggest that SGLT2 inhibitors and metformin are vital not only for diabetes management but also provide new avenues for treating myocardial fibrosis and metabolic disorders.

The term “inhibition” (intensity 2.86, Fig. 10) is directly linked to drug action mechanisms, reflecting the research trend of targeting glycolytic inhibition. The University of Alberta (15 papers) and Harvard Medical School (10 papers) lead related research (Table 3), with numerous findings published in journals such as Circulation Research. Since 2020, “metabolic reprogramming” and “SGLT2 inhibitors” have shown a close association (Fig. 11), highlighting the acceleration of clinical translation research. SGLT2 inhibitors and metformin demonstrate significant anti-fibrotic potential by inhibiting key glycolytic enzymes and modulating metabolic pathways. Future efforts should expand clinical trial scales and explore combination drug strategies to enhance efficacy.

#### Interdisciplinary technologies and future directions

Research on myocardial fibrosis and glycolysis is quickly expanding into interdisciplinary fields. Integrating single-cell sequencing, artificial intelligence (AI), and gene editing provides new tools for understanding mechanisms and developing treatments.

Krstevski et al. reviewed how single-cell RNA sequencing (scRNA-seq) uncovers novel cell types and molecular mechanisms in myocardial fibrosis, offering crucial insights into its cellular basis [85]. Gladka et al. employed single-cell RNA sequencing to study transcriptomic features in healthy and diseased hearts, discovering the regulatory role of cytoskeleton-associated protein 4 in myocardial fibrosis. This underscores the potential of single-cell technology in revealing unknown molecular roles in heart disease [86]. Rao et al. integrated single-cell RNA sequencing and microarray data to explore macrophage infiltration in heart failure. They utilized AI and machine learning algorithms to identify key genes related to immune responses, advancing interdisciplinary technological integration and innovation [87]. Kattih et al. employed single-nucleus transcriptome analysis to reveal the activation of persistent fibroblasts in chronic heart disease patients, further demonstrating the broad application of single-cell technology in fibrosis research [88]. Methatham et al. introduced a concept combining glycolysis with TGF-β and KLF5 signaling pathways, exploring gene editing and metabolic intervention’s potential in treating myocardial fibrosis, providing a theoretical basis for future therapeutic strategies [75].

Existing reviews discuss single-cell or AI technologies in cardiovascular research but fail to link them to the glycolysis-fibrosis axis. Our originality lies in quantifying how these technologies are reshaping the field specifically for glycolysis-related fibrosis research. For example, we find that post-2020, studies incorporating “single-cell sequencing” are 2.1-fold more likely to focus on “fibroblast metabolic heterogeneity” (a key gap in prior glycolysis research) than pre-2020 studies. Additionally, AI-driven “in silico drug screening” (a keyword with rising burst intensity since 2022) shows a 40% co-occurrence with “glycolytic inhibitors”—evidence that technology is accelerating translational research in this niche. This targeted analysis of technology-fmechanism links is absent in prior work, making our findings a unique resource for researchers seeking to leverage interdisciplinary tools.

The emergence of the terms “metabolic reprogramming” (post-2020) and “machine learning” (2022) reflects a trend of technological integration (Figs. 10 and 11). Cluster #1 (systems biology) includes the keywords “machine learning” and “drug discovery” (Table 7), underscoring the interdisciplinary research framework. Since 2020, “single-cell sequencing” and “CRISPR” have shown a close association (Fig. 12), indicating the deepening application of these technologies. Our analysis reveals a paradigm shift toward integrating single-cell sequencing and AI in fibrosis-glycolysis research (Fig. 12), which contrasts with traditional reductionist approaches. This shift offers unprecedented resolution: single-cell RNA-seq uncovers metabolic heterogeneity in cardiac fibroblasts (e.g., glycolytic subpopulations driving ECM deposition), alongside predictive power—machine learning algorithms identify “glycolytic reprogramming” as a hub linking oxidative stress and fibrosis (Fig. 8), enabling in silico drug screening, an advance beyond qualitative reviews.

### Validation of keyword clustering

A key strength of this study lies in the combined use of quantitative clustering metrics and expert validation to ensure the thematic relevance of the keyword clusters. While the Silhouette Value (0.7628) and Modularity Q (0.5292) indicated robust statistical clustering, we further engaged domain experts to evaluate the coherence and accuracy of each cluster label and composition. For instance, Cluster #0 (“pulmonary arterial hypertension”) was confirmed to encapsulate key concepts such as Warburg effect, ventricular ischemia, and pulmonary artery remodeling—all well-established in the literature. Similarly, Cluster #10 (“mitochondrial dysfunction”) was validated to encompass energy metabolism and diabetic cardiomyopathy, reflecting its central role in metabolic heart disease. This expert review mitigates the risk of over-reliance on algorithmic outputs and ensures that the clusters are not only statistically sound but also conceptually meaningful.

## Limitations

This study has several limitations that should be considered when interpreting the results. First, regarding database selection, the analysis was restricted to articles indexed in WOS, Scopus, and PubMed. Although this is a standard and comprehensive approach for international bibliometrics, we did not include Embase or regional databases such as CNKI. The exclusion of Embase was based on its significant content overlap with Scopus, suggesting a limited marginal gain for the substantial additional data processing effort. The exclusion of CNKI was necessitated by the technical incompatibility of its data format with the bibliometric tools used, which require standardized field structures for accurate network analysis. Consequently, some studies, particularly those published in non-English languages or in regional journals, may be underrepresented. Despite this, our data clearly identified China as the second-largest contributing country, indicating that the primary research trends and key contributors from China are effectively captured through international publication channels. Second, the search strategy excluded articles related to cancer. However, some studies on cancer metabolism may discuss mechanisms relevant to myocardial fibrosis. Excluding these articles might result in the omission of important insights. Third, inconsistent terminology across studies, such as “glycolytic reprogramming” versus “metabolic reprogramming,” may decrease the resolution of co-occurrence analyses. Finally, publications from 2024 may have lower citation counts due to the limited time for citations to accumulate, which could underestimate their impact. It is important to specify the time cut-off (December 2024) in the discussion and to note that emerging topics from 2023 to 2024, such as extracellular matrix–metabolism interactions, require additional validation.

## Conclusion

This bibliometric analysis synthesized 408 publications on myocardial fibrosis and glycolysis from 2000 to 2024 to clarify the field’s landscape. Key findings: (1) The U.S. and China lead international collaboration, contributing 32% and 20% of global publications respectively—the U.S. focuses on mitochondrial metabolism, China on epigenetic regulation and clinical translation; (2) Current research centers on glycolytic reprogramming (targeting HIF-1α/HK2/LDHA axis), oxidative stress modulation (NLRP3 inflammasome interactions), and metabolic interventions (SGLT2 inhibitors, metformin), with these pathways in preclinical/early clinical validation for anti-fibrotic drug development; (3) Emerging frontiers include mitochondrial dysfunction (linking cardiomyocyte-fibroblast metabolic competition to fibrosis) and extracellular matrix remodeling (lactate-driven histone lactylation regulating collagen deposition), plus pulmonary hypertension as a cross-disease direction due to shared glycolytic dysregulation.

Practical guidance: (1) Clinical translation: Prioritize fibroblast-specific glycolytic inhibitors and validate SGLT2 inhibitors combined with traditional anti-fibrotic agents for heart failure with preserved ejection fraction; (2) Technical approaches: Integrate single-cell RNA sequencing, multi-omics, and AI for glycolysis-targeted small molecule screening to accelerate precision therapy; (3) Interdisciplinary expansion: Explore biomechanics-metabolomics intersections and shared glycolytic pathways in myocardial fibrosis and pulmonary hypertension.

This analysis confirms glycolysis as a core regulatory axis in myocardial fibrosis. Targeting glycolytic reprogramming and its downstream effects enables precise anti-fibrotic strategies, improving prognosis for fibrotic cardiovascular diseases like post-myocardial infarction and diabetic cardiomyopathy. While limitations such as English-database reliance need addressing, identified trends provide a data-driven framework for basic research and clinical translation.

## Data Availability

The authors will make the raw data supporting the conclusions of this article available, without any undue reservation.

## Glossary of key terms

Note: Usage Context” specifies whether the term is primarily used in the search strategy (Search Term), result interpretation (Interpretation Term), or both. “Associated Terms” links synonyms or conceptually related terms to avoid confusion.

**Table:**

Term	Definition	Usage Context	Associated Terms & Notes
Aerobic Glycolysis	Glycolytic pathway that occurs in the presence of oxygen (e.g., the Warburg effect in fibroblasts), producing lactate and ATP.	Both	Search Term: Used in database queries (e.g., PubMed/Scopus); Interpretation Term: Describes fibroblast metabolic reprogramming in fibrosis.
Bibliometric Analysis	Quantitative analysis of publication data (e.g., publications, citations, keywords) using mathematical/statistical methods to map research landscapes.	Interpretation Term	Methods: Implemented via CiteSpace/VOSviewer; Focuses on trends, collaborations, and hotspots.
Burst Intensity	A bibliometric metric (CiteSpace) measuring the rate of sudden increase in a keyword’s citation frequency, indicating emerging research frontiers.	Interpretation Term	Applied to identify trends (e.g., “extracellular matrix” burst intensity = 3.01, 2021–2024).
Myocardial fibrosis	Pathological process of excessive extracellular matrix (ECM) deposition in cardiac tissue, including myocardial and interstitial fibrosis.	Both	Search Term: Synonym for “myocardial fibrosis” in database queries; Interpretation Term: Core pathological outcome linked to glycolysis.
Cardiac Remodeling	Structural/functional changes in the heart (e.g., ventricular hypertrophy, ECM deposition) induced by pathological stimuli (e.g., pressure overload).	Both	Search Term: Included in fibrosis-related keywords; Interpretation Term: Linked to glycolytic dysregulation (e.g., cluster #2 “cardiac hypertrophy”).
Centrality	A network metric (VOSviewer/CiteSpace) evaluating a node’s (country/keyword) importance in connecting other nodes; values > 0.1 indicate key nodes.	Interpretation Term	Applied to validate core countries (U.S. centrality = 0.28) and keywords (“cardiac hypertrophy” centrality = 0.25).
Co-occurrence Analysis	Bibliometric method to identify relationships between entities (e.g., keywords, authors) that appear together in the same document.	Interpretation Term	Used for keyword clustering (e.g., “glycolysis” co-occurs with “metabolism”) and collaboration networks.
Embden-Meyerhof Pathway	The classical biochemical pathway of glycolysis, converting glucose to pyruvate/lactate.	Search Term	Synonym for “glycolysis”; Used in database queries to ensure comprehensive retrieval (avoids missing niche terminology).
Extracellular Matrix (ECM)	Complex network of proteins (e.g., collagen) secreted by fibroblasts; excessive deposition drives myocardial stiffness in fibrosis.	Interpretation Term	Linked to glycolysis via lactate-induced lactylation (e.g., burst keyword “extracellular matrix,” 2021–2024).
Glycolysis	Core metabolic pathway producing ATP via glucose breakdown; abnormal activation promotes fibroblast proliferation in fibrosis.	Both	Search Term: Core keyword for metabolic queries; Interpretation Term: Focus of “glycolysis-fibrosis axis” mechanistic analysis.
Glycolytic Reprogramming	Pathological shift in cell metabolism toward increased glycolysis (e.g., fibroblasts in fibrosis adopting the Warburg effect).	Interpretation Term	Derived from search term “glycolysis”; Describes mechanistic changes (e.g., HIF-1α-driven HK2/LDHA upregulation).
Hypoxia-Inducible Factor-1α (HIF-1α)	Transcription factor activating glycolytic enzyme expression (e.g., HK2, LDHA) under hypoxia/inflammation, linking glycolysis to fibrosis.	Interpretation Term	Key mechanism connecting search terms “myocardial fibrosis” and “glycolysis” (e.g., Sect. 1 Background).
Myocardial Fibrosis	Fibrotic remodeling specifically in myocardial tissue, a terminal pathological outcome of cardiovascular diseases (e.g., myocardial infarction).	Both	Primary search term (e.g., WOS query: “myocardial fibrosis” AND “glycolysis”); Interpretation Term: Core disease focus.
Myocardial Interstitial Fibrosis	Subtype of myocardial fibrosis characterized by ECM deposition in myocardial interstitium, impairing diastolic function.	Search Term	Included in search strategy to capture subtype-specific studies; Interpretation Term: Linked to “extracellular matrix remodeling” (cluster #0).
Warburg Effect	Aerobic glycolysis in non-cancer cells (e.g., fibroblasts) characterized by lactate production despite adequate oxygen; drives fibrosis progression.	Both	Search Term: Included to capture metabolic reprogramming studies; Interpretation Term: Describes fibroblast metabolism (Sect. 1 Background).

## Standardization notes for term consistency

To ensure alignment between search strategy terms and interpretation terms, we have standardized the following key associations (also summarized in Table 7):

1. Fibrosis-Related Terms (Search → Interpretation).

Search terms (e.g., “cardiac remodeling,” “myocardial interstitial fibrosis”) are mapped to interpretation-focused concepts:

“Cardiac remodeling” → “cardiac hypertrophy + ECM deposition” (cluster #2, #6);

“Myocardial interstitial fibrosis” → “extracellular matrix remodeling” (burst keyword, 2021–2024).

2. Glycolysis-Related Terms (Search → Interpretation).

Search terms (e.g., “Embden-Meyerhof pathway,” “aerobic glycolysis”) are unified under “glycolysis” or “glycolytic reprogramming” in interpretations:

“Embden-Meyerhof pathway” → “glycolysis” (biochemical pathway synonym);

“Aerobic glycolysis” → “Warburg effect in fibroblasts” (mechanistic description).

3. Methodological Terms.

Terms like “centrality” and “burst intensity” are consistently defined using CiteSpace/VOSviewer’s standard metrics (no alternative definitions used);

Clustering labels (e.g., #0 “pulmonary arterial hypertension”) are validated via expert review to ensure alignment with both search terms (e.g., “pulmonary hypertension”) and interpretation (e.g., glycolytic dysregulation in pulmonary hypertension).

This glossary and standardization framework ensure that terms used in database retrieval (search strategy) and scientific interpretation (results/discussion) are consistent, transparent, and accessible to readers across disciplines (e.g., cardiology, metabolomics, bibliometrics).

## Abbreviations

ECM: Extracellular matrix
FAO: Fatty acid oxidation
HIF-1α: Hypoxia-inducible factor-1α
GPR81: G protein-coupled receptor 81
LSI: Latent Semantic Indexing
HK2: Hexokinase 2
PKM2: Pyruvate kinase M2
TG2: Transglutaminase 2
BGN: Biglycan
EndMT: Endothelial-mesenchymal transition
